# Reticulated Origin of Domesticated Emmer Wheat Supports a Dynamic Model for the Emergence of Agriculture in the Fertile Crescent

**DOI:** 10.1371/journal.pone.0081955

**Published:** 2013-11-29

**Authors:** Peter Civáň, Zuzana Ivaničová, Terence A. Brown

**Affiliations:** 1 Manchester Institute of Biotechnology, Faculty of Life Sciences, University of Manchester, Manchester, United Kingdom; 2 Department of Genetics, Faculty of Natural Sciences, Comenius University, Bratislava, Slovakia; 3 Centre of Marine Sciences, University of Algarve, Faro, Portugal; 4 Centre of the Region Haná for Biotechnological and Agricultural Research, Institute of Experimental Botany, Olomouc, Czech Republic; United States Department of Agriculture, United States of America

## Abstract

We used supernetworks with datasets of nuclear gene sequences and novel markers detecting retrotransposon insertions in ribosomal DNA loci to reassess the evolutionary relationships among tetraploid wheats. We show that domesticated emmer has a reticulated genetic ancestry, sharing phylogenetic signals with wild populations from all parts of the wild range. The extent of the genetic reticulation cannot be explained by post-domestication gene flow between cultivated emmer and wild plants, and the phylogenetic relationships among tetraploid wheats are incompatible with simple linear descent of the domesticates from a single wild population. A more parsimonious explanation of the data is that domesticated emmer originates from a hybridized population of different wild lineages. The observed diversity and reticulation patterns indicate that wild emmer evolved in the southern Levant, and that the wild emmer populations in south-eastern Turkey and the Zagros Mountains are relatively recent reticulate descendants of a subset of the Levantine wild populations. Based on our results we propose a new model for the emergence of domesticated emmer. During a pre-domestication period, diverse wild populations were collected from a large area west of the Euphrates and cultivated in mixed stands. Within these cultivated stands, hybridization gave rise to lineages displaying reticulated genealogical relationships with their ancestral populations. Gradual movement of early farmers out of the Levant introduced the pre-domesticated reticulated lineages to the northern and eastern parts of the Fertile Crescent, giving rise to the local wild populations but also facilitating fixation of domestication traits. Our model is consistent with the protracted and dispersed transition to agriculture indicated by the archaeobotanical evidence, and also with previous genetic data affiliating domesticated emmer with the wild populations in southeast Turkey. Unlike other protracted models, we assume that humans played an intuitive role throughout the process.

## Introduction

 Shortly after the Younger Dryas (12,800–11,600 BP) – the closing cold and dry echo of the last glaciation – the nomadic hunter-gatherer communities of southwest Asia adopted a sedentary lifestyle. The reasons for this cultural innovation and the accompanying changes in subsistence strategy have been widely debated, with underlying causes sought among factors as diverse as labor productivity [1], climatic response [2,3], predator-prey relationships [4,5], human intuition [6] and a changing human worldview [7]. Whatever the drivers, the beginning of agriculture was a central component of the set of changes associated with the Neolithic, and is viewed as the major transition in the human past, the period when humans first began to exert a degree of control over their food resources [8].

 Agricultural origins in southwest Asia are traditionally associated with eight founder crops including three cereals, einkorn wheat (*Triticum monococcum* L.), emmer wheat (*T. turgidum* L.) and barley (*Hordeum vulgare* L.) [9]. Archaeobotanical and genetic analysis of these crops, especially the cereals, is increasingly being used as a means of studying the human dimension to the adoption of agriculture [8]. Initially, much of this work was influenced by experimental studies which showed that if appropriate husbandry practices were applied, then the period required for a wild cereal to undergo the suite the phenotypic changes associated with domestication might be as short as a few decades [10]. The attractive idea that a single group of enlightened people could have been responsible for the domestication of one or more staple crops within a few human generations [11] was supported by the first comprehensive genetic comparison of wild and cultivated cereal genotypes [12], which was interpreted as indicating a rapid domestication of einkorn in the Karaca Dağ region of southeast Turkey [6,13,14]. Attempts to extend this rapid, localized model to other crops were initially successful [15], but the paradigm was challenged by computer simulations which showed that the tree-building algorithms used to analyze the genetic datasets could not distinguish crops that are truly monophyletic from ones resulting from multiple independent domestications [16,17]. Archaeological research also began to provide conflicting evidence in the form of archaeobotanical data suggesting that cereal domestication was a protracted process that began with a lengthy period of wild plant management before a slow and piecemeal emergence of the domestication phenotypes, the whole process taking several millennia [8,18,19].

 The conflict between these opposing views of the origins of agriculture is exemplified by the work carried out with emmer and other tetraploid wheats. In the first major genetic study, Özkan et al. [20] used distance-based tree building to compare variations at 204 amplified fragment length polymorphisms (AFLPs) in 43 domesticated lines and 99 wild populations, and identified a single origin for tetraploid wheat domestication near Karaca Dağ. A subsequent examination of chloroplast microsatellite haplotypes, including accessions from areas neglected in the AFLP study, concluded that emmer was domesticated in the northwestern edge of the Fertile Crescent (referred to as Kartal Daği by the authors), some 250 km west of Karaca Dağ [21]. However, two distinct chloroplast lineages were identified in the domesticated plants, suggesting at least a biphyletic origin. AFLPs were then analyzed in the additional wild accessions [22], but the results did not confirm the chloroplast data and instead located the closest wild relatives of domesticated emmer in the Karaca Dağ and Sulaymaniyah (Iraq/Iran border) regions. Luo et al. [23] attempted to solve the puzzle by analyzing restriction fragment length polymorphisms (RFLPs) in 227 wild and 241 domestic tetraploid wheats. A significant proportion of the domesticated lines showed equally strong genetic affinity with wild populations from the Diyarbakir region and southern Levant, leading the authors to conclude that emmer was either domesticated independently in these two regions, or was domesticated in the Diyarbakir region and subsequently acquired additional diversity by gene flow from wild populations in the southern Levant and other parts of the Fertile Crescent. The latter model was supported by a reanalysis of the AFLP data [24], but the former – separate domestications in Turkey and the Levant – agrees with the outcome of an independent study of glutenin alleles in 185 domestic and 59 wild tetraploid wheats [25].

 The contradictory scenarios arising from the genetic analyses contrast with the outcomes of archaeobotanical studies. Preserved emmer spikelets with rough abscission scars indicative of nonshattering ears, looked on as the key domestication phenotype [26], appear simultaneously in the pre-pottery Neolithic B (PPNB, c.10,000 BP) layers of archaeological sites in the southern Levant (Jordan Valley), northern Syria and southeast Turkey [15,24,27,28]. This first emergence of the domestication phenotype was, however, merely one step in the process that led to the fully domesticated crop [8]. For at least 1000 years previously, wild emmer had been cultivated in both the southern and northern Levant [18,29], as revealed by stored assemblages that contain the seeds of weeds associated with arable cultivation. Furthermore, after their first appearance in the archaeobotanical record, the domestication traits rise to dominance only slowly, with different phenotypes following independent dynamics over a period of some 3000 years, in parallel in different parts of the Fertile Crescent [18,26,27,30].

 The above summary of the outcomes of research into the origins of domesticated emmer raises a question which, put bluntly, is why do different genetic analyses of a single crop give such inconsistent results, and why do none of these results agree with the archaeobotanical evidence? Part of the problem lies with the assumption, implicit in the use of phylogenetic methods to analyze genetic data from modern crops, that the evolution of those crops since domestication has been treelike, when in reality there is likely to have been gene flow and hybridization between different crop lineages [16–18]. A second issue that has been less explored is the possibility that domesticated crops have a reticulate rather than linear relationship with their wild progenitor populations. Reticulation refers to the pattern arising when different parts of a genome have different genealogical histories due, for example, to introgression, incomplete lineage sorting (syn. deep coalescence), or hybrid speciation [31]. If a genetic dataset contains incongruent signals resulting from these processes, then a network rather than a tree is a more appropriate representation of the genealogy [32,33]. Should a dataset used to study the origins of domesticated emmer contain such incongruent signals, then these will be suppressed if forced into a single tree, which will show only a single scenario of a pseudo-divergent genealogy. The tree will therefore hide the incompatible signals and not provide the correct interpretation of the domestication process, and different sets of accessions and genetic markers will yield different phylogenetic results.

 To re-assess the origins of domesticated emmer we developed a novel typing method based on detection of DNA polymorphisms associated with the insertion of long terminal repeat (LTR) retrotransposons in the repetitive 5S and 5.8S ribosomal RNA (rRNA) gene arrays. Arrays of 5S rRNA gene-spacer units are located on homeologous wheat chromosomes 1 and 5 [34], each array containing thousands of units [35]. The 5.8S rRNA genes lie within the main multicopy rDNA arrays, which are located independently of the 5S arrays on wheat chromosomes 1A, 1B and 6B [36]. Individual rDNA units are not subject to selective pressure [37], allowing the accumulation of mutations including transposable element insertions.

 To obtain independent evidence regarding the relationship between wild and domesticated emmer, we also re-examined previously-reported sequence data [38] for 21 loci in tetraploid wheats. Based on a tree constructed from the concatenated sequence data matrix, the previous report concluded that domesticated emmer has a monophyletic origin. We show that this conclusion lacks statistical support because of extensive gene-tree conflicts. With both the retrotransposon and nuclear gene datasets we examine the scale of the phylogenetic incongruence with the aid of filtered supernetworks [52] and interpret the phylogeographical data with respect to the revealed reticulation. The results enable us to propose a dynamic model for agricultural origins based on a human driven dispersal of wild plants prior to domestication. The model offers an explanation for the observed patterns of diversity and reticulation, is consisted with the archaeological evidence for domestication as a protracted and dispersed process, and assigns an active role to the early farmers in shaping the geographic distribution and genetic constitution of emmer wheat.

## Materials and Methods

### Wheat Samples and DNA Extraction

 The sample set (Table A in File S1) comprised 227 accessions including tetraploid wheats with both the BA^u^ and GA^u^ genome constitutions. The former, covering all geographical regions, were 70 wild emmers (*T. turgidum* L. subsp. *dicoccoides* (Korn. ex Asch. & Graebn.) Thell.), 99 hulled domesticates (*T. turgidum* L. subsp. *dicoccum* (Schrank ex Schübl.) Thell., *T. ispahanicum* Heslot, *Triticum turgidum* L. subsp. *paleocolchicum* Á. & D. Löve), and 36 free-threshing domesticates including *T. turgidum* subsp. *durum* (Desf.) Husn. and examples of rarely analyzed ancient subspecies of *T. turgidum* (subsp. *carthlicum* (Nevski) Á. & D. Löve, subsp. *turanicum* (Jakubz.) Á. & D. Löve, subsp. *turgidum*, subsp. *polonicum* (L.) Thell.). The GA^u^ tetraploids comprised 16 wild accessions (*Triticum timopheevii* (Zhuk.) Zhuk. subsp. *armeniacum* (Jakubz.) Slageren) and 6 domesticates (*Triticum timopheevii* (Zhuk.) Zhuk. subsp. *timopheevii*). Maps depicting the geographical locations of accessions were drawn with ArcGIS 10 (ESRI).

 To extract DNA, 2–5 grains were crushed to powder and mixed with approximately 400 µl extraction buffer (100 mM Tris-HCl, 20 mM Na_2_EDTA, 1.4 M NaCl, 2% cetyl trimethylammonium bromide, 0.3% (v/v) β-mercaptoethanol) in a 2.0 ml tube. After 1 h incubation at 65°C, samples were centrifuged for 2 min at 13,000×*g* and the supernatant mixed in a 1:5 ratio with binding buffer (High Pure PCR Product Purification Kit, Roche). Extracts were purified according to the manufacturer's instructions and DNA quality and quantity assessed by electrophoresis in 1% agarose gels.

### Genotyping of LTR Retrotransposon Insertions in rRNA Gene Arrays

**Figure 1:**
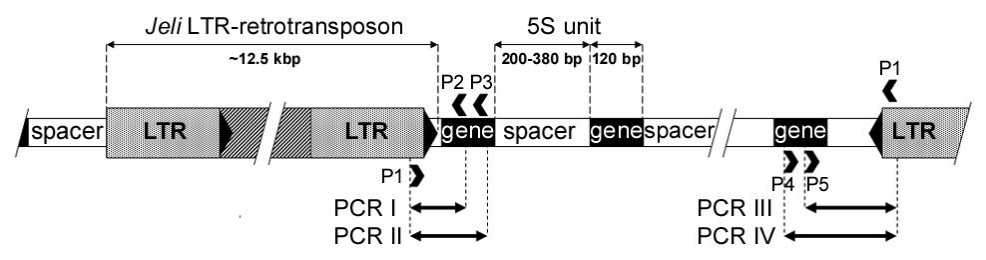
PCR system for detection of LTR retrotransposon insertions in the 5S and 5.8S rDNA loci. The example shown is for detection of a Jeli insertion in a 5S array. Primer P1 is specific for the distal region of the LTR, and primers P2-P5 anneal at different positions with the 5S gene. Depending on its orientation, a Jeli sequence is detected by PCRs with primer combinations P1-P2 and P1-P3, or P1-P4 and P1-P5. Two different PCRs are carried out for each detection to reduce false-positive results. A similar strategy is used to detect other types of LTR retrotransposon and to identify insertions adjacent to 5.8S genes.

 PCR was used to detect polymorphic LTR retrotransposon insertions in 5S rRNA arrays, using combinations of primers specific to the 5S rRNA gene repeat and the LTRs of different classes of wheat retrotransposon (Figure 1). The 5S primers were designed by identifying conserved sequences in the wheat 5S genes present in GenBank, avoiding possible cross-annealing to *Cassandra* retrotransposons which carry a 5S gene-like sequence in their LTRs [39]. The LTR primers were designed from wheat retrotransposons present in the TREP database [40]. LTR sequences were aligned and family-specific annealing sites identified for outward facing primers. A similar method was used to detect retrotransposon insertions in the vicinity of 5.8S rRNA genes within the main rDNA arrays. Primer design was aided by FastPCR [41] and Primer-BLAST (www.ncbi.nlm.nih.gov/tools/primer-blast/); primer sequences are listed in Table B in File S1. Preliminary tests on a small set of emmer samples showed that PCRs with LTR primers specific for the *Jeli* and *BARE1*/*Wis*/*Angela* retrotransposon families gave polymorphic products of the expected size range. Since it is known that the *Jeli* and *Angela* families have been actively transposing since the formation of tetraploid wheat [42], the latter primer was further adjusted to detect only *Angela* retrotransposons. The specificity of PCRs with this primer was checked by sequencing products of different lengths obtained from several einkorn samples.

 The tetraploid sample set was genotyped with six primer combinations targeting *Jeli* and *Angela* insertions (Table B in File S1) in 12.5 µl PCRs containing 1 x reaction buffer, 1.75 mM MgCl_2_, 0.25 μM each primer, 0.2 mM each dNTP, 0.025 U/μl AmpliTaq Gold DNA polymerase (Applied Biosystems) and ~30 ng template DNA. Cycling conditions were: 5 min at 95°C; 35 cycles of 45 s at 95°C, 45 s at 57°C, 30 s at 72°C; 7 min at 72°C. All PCR products were analyzed by electrophoresis for 1.5 h at 5V/cm in 1.75% agarose gels; the products of *Jeli* PCR-screening were additionally genotyped using an Applied Biosystems 3730 DNA Analyzer with 500 LIZ size standard to resolve alleles of similar size.

 Products of the *Jeli*–5.8S PCR, which were of uniform size (~720 bp), were sequenced (Macrogen) for 186 accessions (GenBank accession numbers JX470351-JX470368). These sequences were aligned with Geneious 5.1.7 [43] and a MJ network constructed in Network 4.6 [44]. Each *Jeli*-5S and *Angela*-5S amplicon of distinct size was regarded as an independent insertion. Insertions detected in only one sample were discarded as phylogenetically uninformative, and a few markers that were difficult to score (due to poor amplicon synthesis for some accessions) were omitted. The remaining insertions were depicted as a virtual gel in Geneious.

 The sequence and binary retrotransposon markers were also analyzed in combination. The *Jeli*-5.8S alignment was dissolved into individual polymorphic positions (splits) and all such splits imported into SplitsTree4 [45] as newick tree vectors. Individual binary markers of the corresponding sample set were loaded into SplitsTree4 in the same way and the resulting set of 43 splits used to construct a filtered supernetwork [32]. The minSupportingTrees parameter was set to 31, meaning that only the splits congruent with >31 other splits are shown in the network. This reduction of network distortion is expected to filter out conflicting ‘noise’ resulting from splits which may be present in the input datasets due to homoplasy and deep coalescence [33,46]. Other parameters of the supernetwork construction were left at the default values.

### Supernetwork Analysis of Published Sequence Data

 Sequence data from 21 loci (*11B*, *91A*, *AapA*, *AlperA*, *Bp3B*, *Bp2A*, *Bp5A*, *ChsA*, *Gsp1A*, *Gsp1B*, *HgA*, *HiplA*, *MdhA*, *Mdh4B*, *Mp7A*, *MybA*, *MybB*, *NrpA*, *PsyA*, *ZdsB*) of tetraploid wheats (*T. turgidum* subsp. *dicoccoides*, *dicoccum*, *durum*; *T. timopheevii*) published by Haudry et al. [38] were downloaded from GenBank, recoded according to geographic origin (Table C in File S1) and aligned in Geneious. The sequence alignments were edited by extracting polymorphic positions, removing sequences or columns with multiple unrecognized bases, and reducing indels >1 bp to single positions. A concatenated data matrix was created from all 21 genes and the most parsimonious (MP) tree was searched in dnapars of PHYLIP 3.67 [47] with 10 jumble runs and the remaining options at the default values. A bootstrap analysis with 1000 resampled datasets was also conducted and a majority-rule consensus tree was constructed. The same approach was used to construct an MP tree for each individual locus. If several equally parsimonious trees were identified for a given gene, a strict consensus tree was constructed. For five loci (*11B*, *91A*, *Mdh4B*, *MybB*, *NrpA*), outgroup data (*T. timopheevii*) were not available. The topology of the trees for these loci could therefore be misleading and confound the supernetwork construction by introducing false incongruence among the gene trees. Therefore, a reduced dataset of 15 loci – omitting the five loci with no outgroup as well as the *ChsA* locus (extensive missing data) – was processed alongside the full dataset consisting of all 21 loci. From the resulting sets of 21 and 15 partial trees (representing 28–65 accessions and 37–65 accessions, respectively) filtered supernetworks were produced in SplitsTree4. The minimal set of partial trees required to support a split (minSupportingTrees parameter) was adjusted to eight and six in the full and reduced datasets, respectively. The remaining settings were left as default. The partial trees from the reduced dataset were also analysed in pairs with the autumn algorithm implemented in Dendroscope 3 [48] to identify conflicting tree pairs and the minimal number of hybridizations required to explain the given conflicts. 

## Results

### Jeli–5.8S Amplicon Sequences

 We typed polymorphic retrotransposon insertions in a collection of tetraploid wheats (Table A in File S1) by carrying out PCRs with combinations of primers that targeted conserved regions within the 5S and 5.8S rRNA genes and the LTRs of different groups of wheat retrotransposons (Figure 1, Table B in File S1). The PCR system comprising one primer specific for the 5.8S gene and one specific for the LTR of the *Jeli* group of retrotransposons gave a product of uniform size (~720 bp), which was sequenced for each of 186 accessions. The first 55 bp of this sequence aligns with the wheat 5.8S gene and the remainder appears to be an atypical LTR sequence.

**Figure 2:**
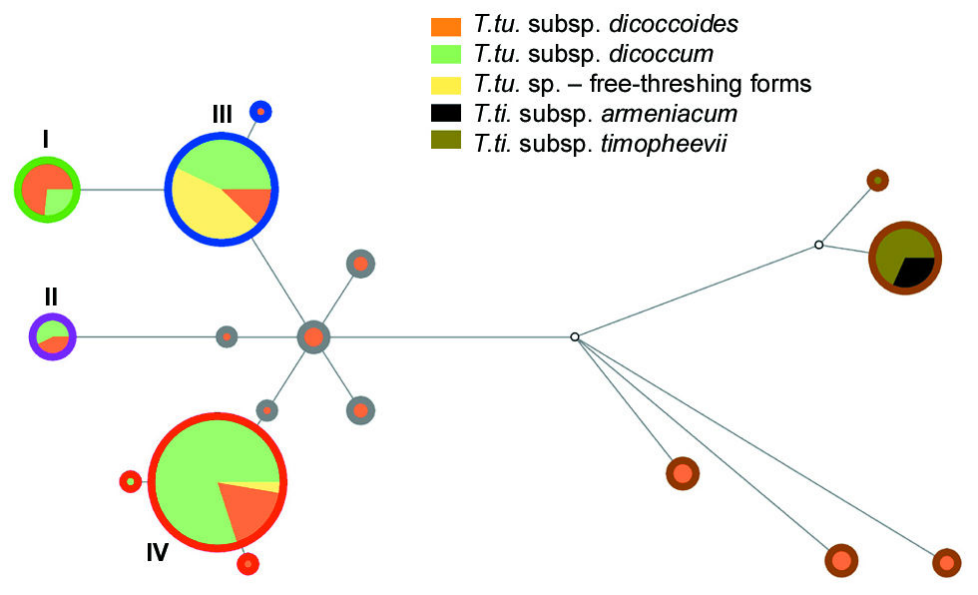
MJ network constructed from 5.8S-*Jeli* sequence data. Node sizes are proportional to the number of accessions displaying that allele, and the edge lengths are proportional to the number of substitutions between pairs of allele sequences. The taxonomic content of each node is indicated as a pie chart. The color coding of the outer circle of each node relates to the symbols used for different groups of accessions in Figure A in File S1.

 Seventeen alleles were identified in the 186 accessions (π = 5.68 × 10^-3^). In a median joining (MJ) network those alleles shared between wild and domesticated forms of *T. turgidum* fell into four distinct groups (clusters I–IV), which made three independent connections with the remainder of the network (Figure 2). Wild emmers with alleles from the smaller clusters I and II are geographically localized in the southern Levant and the NW Fertile Crescent, respectively (Figure A part A in File S1). Those from the larger clusters are geographically dispersed, from the Levant to Iraq/Iran (cluster III), and from the Jordan Valley to Diyarbakir region (cluster IV) (Figure A part B in File S1). 

 The MJ network topology located ten alleles outside of clusters I–IV and hence absent in domesticated emmer. These alleles could be divided in two groups of five, one group comprising alleles basal to the domesticate clusters (circled in gray in Figure 2), and the second group made of more ‘early-diverging’ alleles (circled in brown), the latter including two alleles identified only in *T. timopheevii*. Accessions containing the early-diverging alleles were broadly distributed, with *T. turgidum* in the west arm and *T. timopheevii* in the east arm of the Fertile Crescent, but those *T. turgidum* accessions with basal alleles were restricted to the southern Levant (Figure A part C in File S1). Of the 14 sequence types found in *T. turgidum* subsp. *dicoccoides*, only two were detected in wild emmer from southeast Turkey or Iraq/Iran, and neither of these two alleles were unique to these locations (Figure A part B in File S1). 

### Angela- and Jeli–5S Binary Data

**Figure 3:**
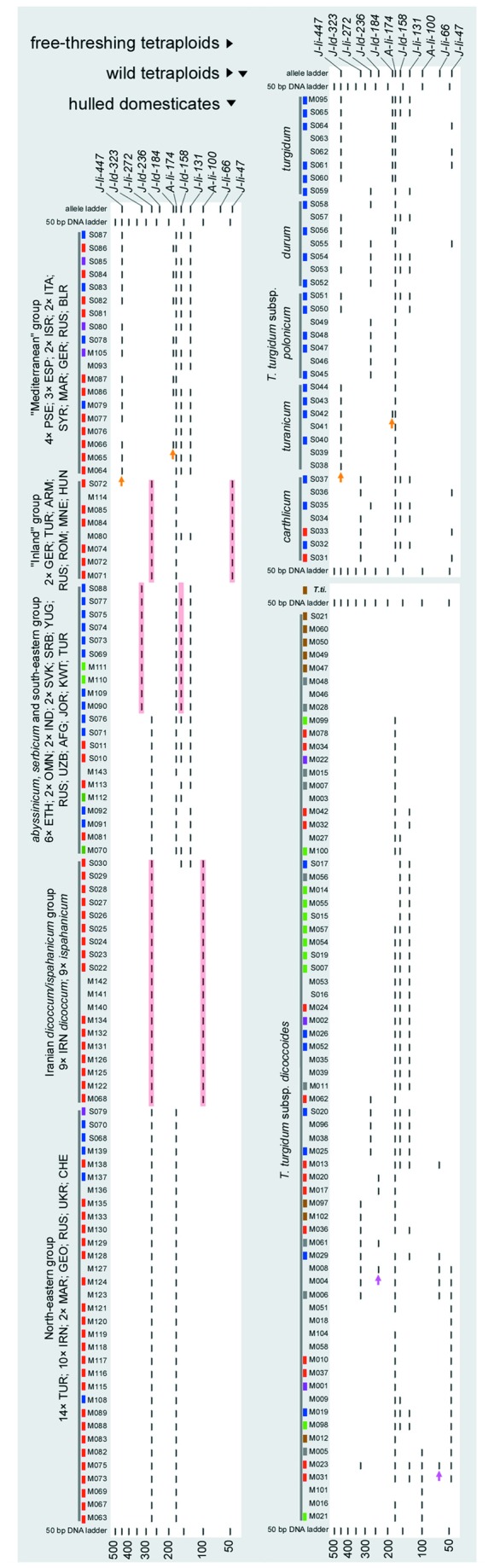
Virtual gel of the retrotransposon insertions detected in the 5S arrays. Accessions are identified by the codes given in Table A in File S1. All *T.timopheevii* accessions displayed the same profile marked here as *T.ti*. Pink and orange arrows indicate insertions detected only in wild and domesticated tetraploid wheats, respectively. Combinations absent in wild populations but frequent in domesticated accessions are highlighted with a pink background. Accessions for which 5.8S-*Jeli* sequences were obtained are color-coded in the same way as the node outer circles in Figure 2.

 We identified eleven distinct insertional polymorphisms of *Angela* and *Jeli* retrotransposons in 5S arrays of tetraploid wheat (designated as *A-Ii-100*, *A-Ii-174*, *J-Id-158*, *J-Id-184*, *J-Id-236*, *J-Id-323*, *J-Ii-47*, *J-Ii-66*, *J-Ii-131*, *J-Ii-272*, and *J-Ii-447*; Figure 3). None of these insertions were present in the *T. timopheevii* and *T. turgidum* accessions with early-diverging *Jeli*–5.8S alleles, implying that the invasion of *Angela* and *Jeli* retrotransposons into 5S arrays has occurred since the emergence of BA^u^ tetraploids, less than 0.5 MYA [49,50]. Typically, an accession gave 1–4 amplicons (<550 bp) with each primer combination (see Figure 1). Two of the *Jeli*–5S insertions (*J-Ii-66* and *J-Id-236*) were present only in wild emmer, and two others (*J-Id-184* and *J-Ii-447*) only in domesticated accessions. The latter presumably originated <10,000 years ago, indicating that *Jeli* retrotransposons are still active in the wheat genome [42]. Among the shared insertions, *J-Ii-272* was present in 71% of the hulled domesticates and 33% of the free-threshing ones, but only in five wild emmer accessions, three of these from the Iranian Zagros mountains. The other shared insertions were present in wild samples from all over the Fertile Crescent (*A-Ii-100*, *J-Id-158*, *J-Ii-131*), or appeared concentrated to the west of the Euphrates (*J-Id-323*, *J-Ii-47*, *A-Ii-100*). Again, the wild emmers from southeast Turkey, Iraq and Iran posses only a subset of variability found in the Levant, missing the markers *J-Ii-47*, *A-Ii-100* and *J-Id-236*. 

 When insertion profiles were considered, clear distinctions were seen between wild and domesticated accessions (Figure 3). For example, the individual insertions *A-Ii-100*, *J-Ii-47*, *J-Id-158*, *J-Ii-272*, and *J-Id-323* were present in both wild and domesticated emmer, but the combinations *A-Ii-100/J-Ii-272, J-Id-158/J-Id-323* and *J-Ii-47/J-Ii272*, although frequent in domesticated emmer (11%, 11%, and 9%, respectively), were not found in wild accessions. The insertion profiles distinguished between the five subspecies of naked tetraploids, although with some signs of gene flow, and divided the hulled emmer domesticates into five broad phylogeographic groups, described as ‘North-eastern’, ‘Iranian *dicoccum*/*ispahanicum*’, ‘*abyssinicum*, *serbicum* and south-eastern’, ‘Inland’ and ‘Mediterranean’.

### Combined Retrotransposon Data

**Figure 4:**
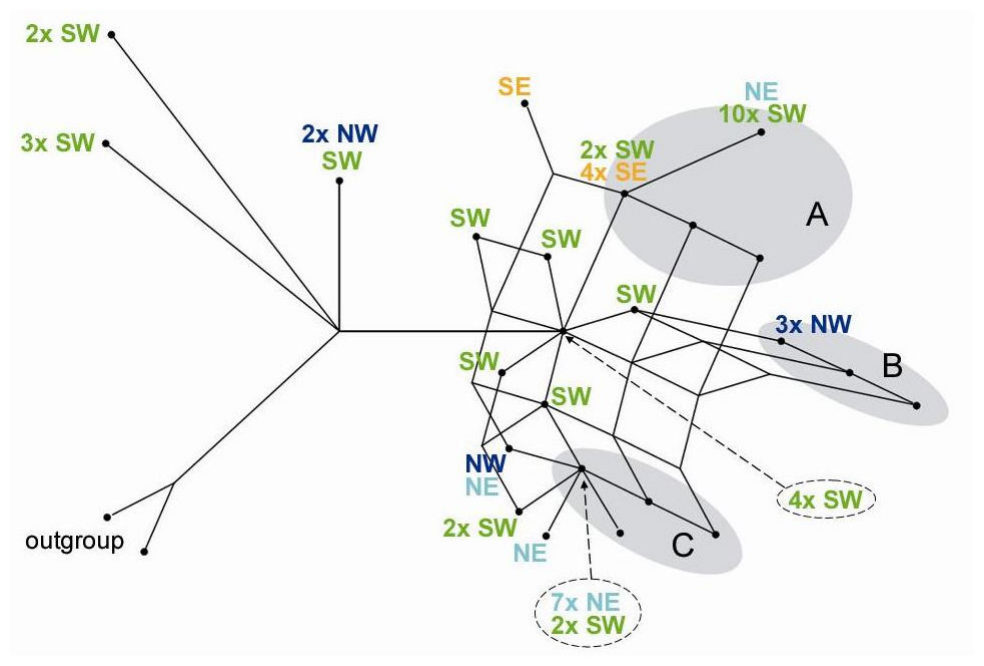
Supernetwork of combined retrotransposon data. Network nodes containing samples are marked by black dots. The geographic locations of the wild emmer accessions are indicated next to the corresponding nodes as follows: *SW* (green), southern Levant (Israel, Lebanon, Jordan, SW Syria); *NW* (dark blue), northwest (western part of the Syria–Turkey border, here approximated as 'Gaziantep region'); *NE* (light blue), northeast (vicinity of Karaca Dağ); *SE* (orange), southeast (parts of Iraq and Iran). Domesticated emmer is found in the nodes within the grey background areas. Cluster A includes all but two free-threshing samples (22) together with *dicoccum* accessions from Ethiopia (6), Turkey (5), Oman (2), India (2), Slovakia (2), former Yugoslavia (1), Morocco (1), Palestine (1) and Germany (1). Cluster B includes four *dicoccum* samples (Palestine, Morocco, Turkey and Scandinavia). Cluster C includes all Iranian *dicoccum* (15) and *T. ispahanicum* (8) accessions, *dicoccum* samples from Turkey (8), Russia (4), Spain (3), Italy (2), Hungary (2), Palestine (2), Jordan (2), Germany (2), Switzerland (1), Armenia (1), Georgia (1), Ukraine (1), Belarus (1), Romania (1), Serbia (1), Israel (1), Morocco (1), Eritrea (1), Uzbekistan (1), and two free-threshing samples. The outgroup comprises the *T. timopheevii* accessions.

 The *Jeli*–5.8S, *Angela*–5S and *Jeli*–5S datasets were combined and conflicting phylogenetic signals analyzed by constructing a filtered supernetwork (Figure 4). The part of the network containing the *T. timopheevii* samples and the early diverging wild emmers was free of reticulation. The majority of wild emmers were included in the reticulated part of the network, together with three clusters of domesticated samples. The first cluster (cluster A) contained all but two of the free-threshing samples (exceptions being two *T. turgidum* subsp. *carthlicum* accessions placed in cluster C) suggesting a single origin for the *durum*, *turanicum, turgidum* and *polonicum* subspecies, in accordance with the reported reduction of nucleotide diversity in the *dicoccum-durum* line [38]. Cluster A also contained hulled tetraploids, mainly *dicoccum* samples from Ethiopia, Turkey, India and Oman as well as the *serbicum* varieties. Most of the wild emmers in cluster A came from the southern Levant and Iraq/Iran, with one accession from Karaca Dağ. The second cluster (B) contained four geographically dispersed *dicoccum* samples closely associated with wild emmer from the Gaziantep region. The third cluster (C) included hulled domesticates from Iran and Transcaucasia, as well as most of the *dicoccum* samples from the Mediterranean and inland Europe. Affiliated wild samples mostly came from the Karaca Dağ region, but also from the Levant. These three clusters were interconnected through deeper hybridization links originating within a core of Levantine wild emmers. 

### Reanalysis of Available Sequence Data

 To obtain independent evidence regarding the relationship between wild and domesticated emmer, we re-examined previously-reported sequence data [38] for 21 loci in *T. turgidum* subsp. *dicoccoides* and *T. turgidum* subsp. *dicoccum* (Table C in File S1). Sequence alignments revealed 55 sequence types for the 21 loci in the domesticated emmers, of which 37 were also identified in wild accessions. As some of the 18 remaining, apparently *dicoccum*-specific sequences might be present in unsampled wild populations, it seems likely that post-domestication divergence has played only a minor role in generating diversity. In most cases, therefore, allelic sequences in domesticated emmer are identical to those in wild emmer, but they often appear in different combinations, a clear sign of reticulation. For example, the EF108894 allele of the *GdhA* gene (which is present in domesticates and wild samples west of the Euphrates) is found with the *Bp2A* (EF108668) allele in six of 12 domesticates, and with the *PsyA* (EF115015) allele in five of these 12, but these combinations are not present in any of the wild accessions. Similarly, one allele of *ZdsB* (EF115121; found in domesticates and wild emmers from Israel) combines with the *MdhA* (EF109064), *Mp7A* (EF109521), *GdhA* (EF108895) and *PsyA* (EF115015) alleles in four, three, three and three *dicoccum* samples, respectively, but none of these combinations were seen in wild emmer.

 Only three loci (*AapA*, *Bp5A* and *MdhA*) were monomorphic in domesticated emmer (Table D in File S1). For two of these (*AapA*, *MdhA*) the apparent monomorphism was associated with low overall genetic diversity. Each of the remaining 18 loci displayed two or more sequence types in the domesticates.

 The MP analysis of the concatenated data matrix utilized 218 parsimony-informative characters and identified a single minimal tree with *durum* and *dicoccum* accessions forming a monophyletic group (Figure B in File S1), similar to that previously reported [38]. However, the score of the most parsimonious tree (652) was 1.73 × higher than the sum of the MP trees computed for each locus individually (377), which is symptomatic of gene-tree conflicts. Subsequent inspection of the 15 rooted gene-trees with the autumn algorithm revealed that 84 out of the 105 possible tree-pairs contained one or more conflicts. On average 3.1 hybridization events per conflicting tree-pair are necessary to explain the observed data. The bootstrap analysis of the concatenated data did not provide any statistical support for monophyletic domestication (<50%; Figure B in File S1). As the portion of the phylogenetically informative characters would seems to be sufficient for resolution of the major clades, and sequence homoplasy is unlikely to play a significant role within the studied evolutionary time-span, we conclude that the absence of statistical support is caused by genetic reticulation of emmer lineages.

**Figure 5:**
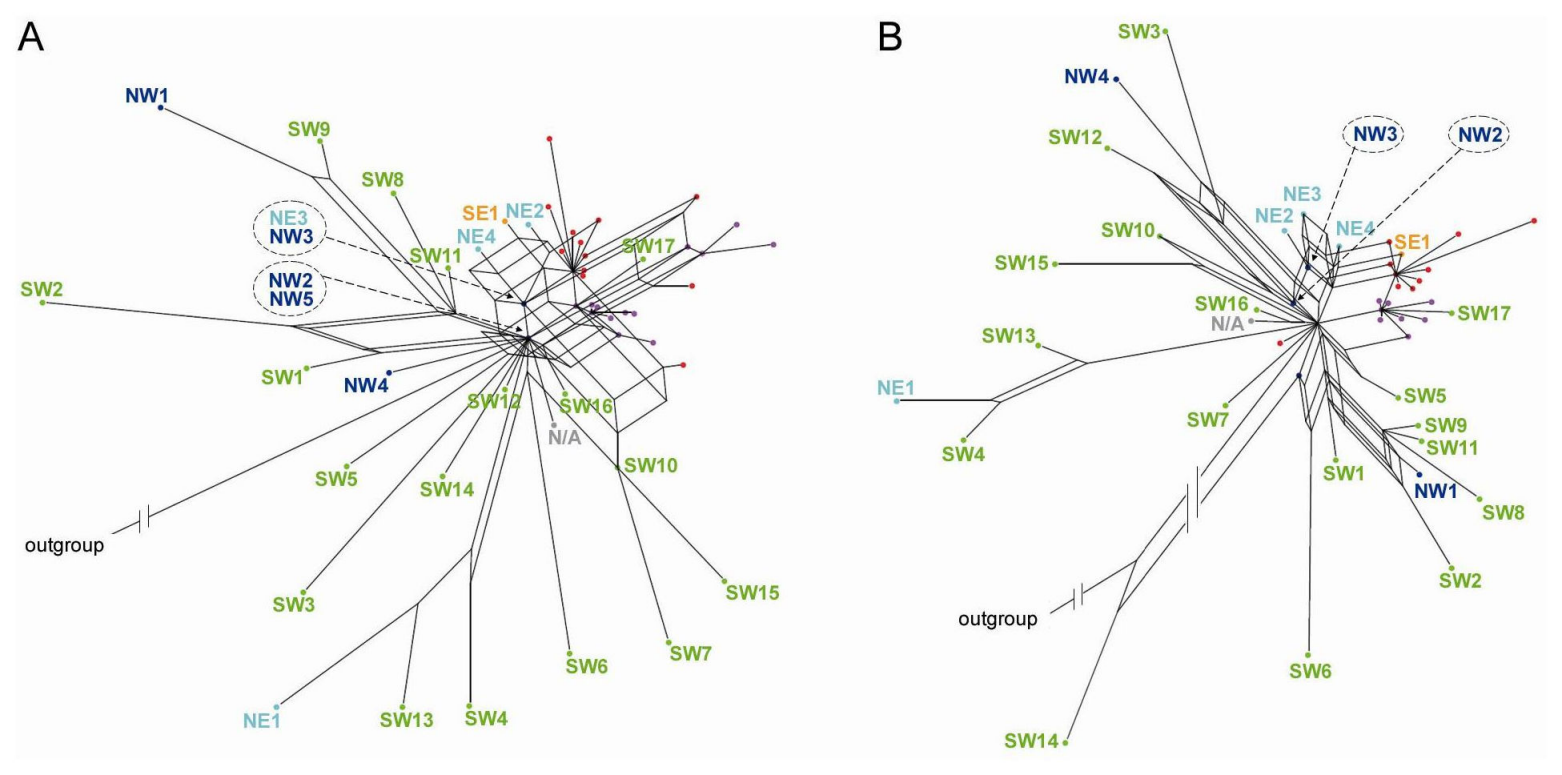
Filtered supernetworks constructed from multiple nuclear gene sequences **[38]**. (A) Full dataset composed of 21 partial trees, and (B) a reduced dataset containing only the 15 rooted partial trees, for sequences from wild emmer (28 accessions, dots color coded according to region, *N/A*, location not available), domesticated emmer (12 accessions, red dots) and *durum* wheat (20 accessions, purple dots). Individual nodes may contain multiple samples. The geographic locations of the wild emmer accessions are indicated in accordance with Figure 4. The outgroup comprises the *T. timopheevii* accessions.

 Filtered supernetworks constructed from these data (Figure 5) confirmed the reticulated origin of *dicoccum* and *durum* wheat. In both supernetworks, *T. turgidum* subsp. *dicoccum* and *durum* accessions are concentrated in two, partially overlapping clusters that mostly consisted of terminal nodes radiating out of reticulated tangles. Similar to the retrotransposon supernetwork (Figure 4), the free-threshing wheats cluster separately from the main *dicoccum* group. The nodes basal to these tangles were typically represented by northern wild emmers and by the central node and other empty nodes related to some of the Levantine wild emmers. The Iranian wild emmer accession (SE1) and three Karaca Dağ samples (NE2, NE3, NE4) also appear to have a highly reticulated origin and occupy terminal rather than basal positions of the tangle associated with the domesticates. This network topology suggests that the eastern wild samples (SE and NE) and domesticated emmer have partially shared hybridization histories.

## Discussion

### Reticulated Ancestry of Domesticated Emmer

 In this study, we identified both sequence and positional polymorphisms for LTR retrotransposon insertions in the 5S and 5.8S arrays of a large set of tetraploid wheat accessions. Size homoplasy of the detected insertion markers is expected to be minimal because it is unlikely that two retrotransposons would insert at exactly the same position in different gene-spacer units. Since these marker loci have virtually no selection value their evolutionary age is likely to be short and deep coalescence of the marker variants will therefore be limited. We believe that these factors, when combined with the known genomic location and relatively clear evolutionary dynamics, provide this typing system with advantages compared to anonymous multilocus markers such as AFLPs, RFLPs and microsatellites. Because of the high sequence conservation of the rRNA genes and the ubiquity of LTR retrotransposons in eukaryotic genomes, the method is likely to be applicable to many species and evolutionary questions.

 Our analysis of retrotransposon polymorphisms in tetraploid wheats showed clear indications of a complex, non-linear relationship between wild and domesticated emmers. These indications included: the presence of four *Jeli*-5.8S allele groups in domesticated emmer, each group also present in wild accessions (Figure 2), a pattern that is inconsistent with a linear monophyletic relationship between the domesticated and wild populations; sharing of individual *Angela*- and *Jeli*-5S insertions, but not combinations of insertions, between wild and domesticated emmers (Figure 3), which is a clear sign of a reticulated relationship between the two populations; considerable incongruence of phylogenetic signals within the supernetwork constructed from the combined retrotransposon data (Figure 4); and the network summary of incongruent phylogenetic signals within the combined retrotransposon dataset, which show that hybridizations are not restricted to solitary samples but instead involve all the domesticates and a large proportion of the wild emmers (Figure 4).

 These results suggest that the origin of domesticated emmer is neither mono- nor polyphyletic, but reticulate, this being the principal reason why the phylogenetic trees previously built from different sets of markers and samples conclude different origins for this crop [20–25]. When considering the evolutionary history of domesticated emmer, it is therefore more meaningful to focus on the geographic distribution of individual phylogenetic signals (e.g. insertion markers and sequence types) rather than the distribution of detected genotypes. Within the domesticated accessions, we have identified phylogenetic signals originating from all the previously reported geographic regions – Gaziantep region, Zagros Mountains, southern Levant and southeastern Turkey. However, the majority of these markers/sequence types are dispersed across broad areas of the Fertile Crescent, and none of them is exclusive to any region east of the Euphrates.

 A reticulate origin for domesticated emmer is supported by our reanalysis of published nuclear gene sequences. Filtered supernetworks constructed from these sequences displayed considerable incongruence in the region containing the domesticated accessions, most likely due to hybridization rather than homoplasy or deep coalescence (Figure 5). The networks show an affiliation between domesticated emmer and northern wild emmers, including some from southeastern Turkey and the Zagros region, as reported previously [20,22,23]. However, the topologies do not indicate that *dicoccum* and *durum* wheats descended from these northern genotypes. Instead they suggest that the domesticates and the northern emmers share a related reticulated ancestry, the northern wild emmers forming a genetic mosaic derived from the same ancestral populations that gave rise to the domesticated cluster. We therefore conclude that domesticated emmer and the northern wild populations have common ancestors west of the Euphrates. 

### Origins of Emmer Cultivation and Possible Impacts on Diversity and Reticulation Patterns

 Previous phylogeographic studies searching for the place(s) of emmer domestication employ an implicit assumption that the distribution of wild emmer populations has not changed substantially since the beginning of the domestication process. Although the possibility that macro- and microclimatic variations might have altered the wild emmer distribution has long been recognized [51], the limited ways of investigating such past changes have meant that this problem has received only marginal attention. However, for the correct interpretation of emmer phylogeny, not only the post-domestication but also the pre-domestication distribution changes may be critical. Our data suggest that the distribution of wild emmer was originally confined to that of the ‘southern race’ [51] in the area of present-day Lebanon, northern Israel and southern Syria, centered around the upper Jordan valley. This is indicated by the topology of the MJ network of *Jeli*-5.8S sequences (Figure 2), in which most of the ‘early-diverging’ wild emmers, which did not contribute to the domesticated gene pool, as well as the wild forms appearing basal to all the cultivated BA^u^ wheats and their immediate ancestors, were collected from this area. A very similar picture, where the basal and early-diverging wild emmers originate from the southern Levant while Turkish and Iranian accessions appear only among the phylogenetically recent nodes, is also suggested by network analysis of 64 published wild emmer *Pm3* gene sequences [52] (Figure C in File S1). These observations suggest that BA^u^ wheat evolved in the southern Levant, as previously suggested [53], or irrespective of origin was restricted by the glaciations to a southern Levant refuge. In either case, the implication is that wild emmer spread to the northern and eastern Fertile Crescent relatively recently.

 For a self-pollinating annual that grows in dense stands, long-distance dispersal does not improve survival [54]. Thus, wild wheat seeds lack features that facilitate wind or animal dispersal. However, wheat seed is easily transported by humans, raising the possibility that human communities contributed to the distribution of wild emmer. According to archaeological evidence, wild emmer has been collected by hunter-gatherers since the Upper Palaeolithic, 23,000 BP [24,29]. For millennia, nomadic communities migrated periodically across the Fertile Crescent, hunting gazelles and other ungulates, harvesting cereals from wild stands and, possibly, carrying grain supplies with them into new territories. Wheat grain is also likely to have featured in the Natufian and PPNA trade network, revealed by archaeological findings of obsidian, that extended in two directions – from Kapadokya (~240 km northwest from Gaziantep) to the southern reaches of the Jordan Valley, and from Bingöl (~150 km northeast from Karaca Dağ) to the Zagros Mountains [55,56]. It is therefore plausible that Epipaleolithic and early Neolithic communities contributed to the spread of wild emmer from its origin in the southern Levant. This scenario is consistent with the 5S- and 5.8S-retrotransposon diversity patterns, which show that the genetic variants possessed by the eastern wild samples represent only a small subset of those present in the Levant. The possibility that wild emmer populations from SE Turkey and Iraq/Iran entered the region only with the first cultivators has been proposed before [28,54], and is supported by the absence of wild emmer at the pre-domestication archaeological sites of northern Syria and southeastern Turkey until 10,500 BP, although einkorn, rye and barley are present prior to this period [24].

 One possible explanation of the reticulations in the evolutionary history of domesticated emmer is that gene flow has occurred between the wild and domesticated populations, leading to introgression of alleles from dispersed parts of the wild population into different components of the crop. Gene flow between wild and domesticated plants has previously been suggested specifically for emmer [23] and more generally for cereals [57], but the scale of its possible impact has been questioned [58]. A second argument against extensive introgression of wild genes into the crop is the possibility that this will result in replacement of the recessive domestication traits, such as the nonshattering ear, with their wild versions, meaning that the introgressed plants are likely to be lost from the domesticated population.

 Our results suggest that the reticulated origins of domesticated emmer are due to gene flow operating within a different scenario. We do not know what husbandry practices were used by the first human communities to cultivate wild cereals [58]. Regardless of whether stands of wild wheat were grown in ‘fields’, it seems likely that the cultivated material evolved into a blend of different populations, resulting from centuries of collection of wild grain from various sources. This mixture of populations would inevitably lead to genetic reticulation in the wild crop as a whole, the extent of this reticulation depending on the frequency of cross-pollination. The frequency of outcrossing in wild emmer has been estimated at 3%, based on the observed heterozygosity deficit [59], or below 1% based on a one-season field experiment [60]. Cross-pollination is enabled by open flowerage, which occurs in humid conditions, especially when coupled with cold, therefore the upper estimate may be more credible as a long term average. In a simple model where two large, genetically distinct emmer populations are intermixed in equal proportions, assuming absence of selection and 1% frequency of cross-pollination, the individuals gradually become a genetic mosaic of the initial two populations (Figure D in File S1). After 200 generations, the frequency of the original genotypes is below 10%, and the pace of these changes is even faster if three populations are intermixed, or more frequent cross-pollination is assumed. Hence, rare random hybridizations can result in relatively complex reticulation, despite the predominantly self-pollinating nature of wheat. We propose that the gene flow leading to the reticulations that we observe in the evolutionary record of domesticated emmer occurred predominantly during the pre-domestication phase, when mixed populations of wild emmer were being cultivated in the southern Levant.

### A Dynamic Model for the Emergence of Agriculture

 According to the scenario described above, the wild emmer of SE Turkey, Iran and Iraq, described as “never really abundant … in sporadic, isolated patches and thin scattered stands” and “hardly have been very attractive to the food-collecting cultures of the region” [51], is descended from the wild emmer grain from the Levant, taken to those more northern regions by humans during the Epipaleolithic and early Neolithic periods. This leads to a model for the origins of domesticated emmer that is consistent both with the previous genetic studies [20–25] and the archaeobotanical evidence for a protracted domestication process [13,57]. We hypothesize that during the Epipaleolithic, the hunter-gatherer communities of the southern Levant began to exercise control over their food supplies by managing wild stands of emmer, these activities progressing to the stage where wild grains were collected and cultivated to provide the next season’s resource. The mobility of these communities engendered a dynamic situation in which distinct wild emmer populations from a large area west of the Euphrates became intermixed in the fields of these early farmers. Hybridization between different lineages within these fields, possibly over an extended period of time, gave rise to pre-domesticated crops that displayed a reticulated evolutionary relationship with the wild populations from which they were originally derived. Accompanying these events, cultivation of wild emmer spread from the southern Levant in a clock-wise direction to other parts of the Fertile Crescent. Movement away from the range of the wild population established reproductive isolation between wild and cultivated plants, facilitating human selection for the domestication traits [5]. The first domesticated plants therefore appeared when cultivation reached northern Syria, southeast Turkey and northern Iraq. The wild emmers found today in the Karaca Dağ and Sulaymaniyah regions, being the remnants of the cultivated population from which the first domesticates evolved, are therefore identified as genetically proximal to the domesticated genepool when phylogenetic methods that enforce a treelike pattern of evolution are used. The genetically domesticated varieties then spread to other farming regions, such that the proportion of domesticated to wild grain at these sites gradually increased. Eventually the domesticated population spread outside the Fertile Crescent, resulting in independent bottlenecks which gave rise to the geographical variability of the tetraploid landraces observed today.

 The protracted model for the origins of agriculture has repeatedly been interpreted as requiring no conscious human involvement in the domestication process [6,58]. Our dynamic model assumes that the transition from hunting-gathering to agriculture was protracted but equally assumes that humans played an active and intuitive role throughout the process. We do not, however, believe that in order to assign humans an active role it is necessary to interpret the transition as a teleological process culminating in conscious human selection of fully domesticated plants. Emergence of the domestication traits might appear, from a retrospective viewpoint, to be the endpoint of a progressive evolutionary process, but the data we present show that, in reality, the genetic changes that underlay ‘domestication’ were merely part of a lengthy series of events that began in the cultivated wild populations, and continued in the domesticated population for centuries after the ‘origin’ of agriculture.

## Supporting Information

File S1 (DOCX)
