# Supplementary material for: Reticulated Origin of Domesticated Emmer Wheat Supports a Dynamic Model for the Emergence of Agriculture in the Fertile Crescent
Source: PLoS One. 2013 Nov 29;8(11):e81955. doi: 10.1371/journal.pone.0081955 (PMC3843696; doi:10.1371/journal.pone.0081955)
Supplement: File S1 — (DOCX) [file pone.0081955.s001.docx]

**Reticulated Origin of Domesticated Emmer Wheat Supports a Dynamic Model for the Emergence of Agriculture in the Fertile Crescent**

**Peter Civáň, Zuzana Ivaničová, Terence A. Brown**

**SUPPORTING INFORMATION**

**Table A** List of wheat accessions used for retrotransposon typing

**Table B** Primer sequences

**Table C** List of wheat accessions reanalyzed from data of Haudry et al.

**Table D** Data obtained by inspection of sequence data for 21 loci in wild and domesticated emmer

**Fig. A** Geographical locations of wild accessions for which *Jeli*–5.8S sequence data were obtained

**Fig. B** The most parsimonious tree constructed from the 21-gene concatenated data matrix.

**Fig. C** MJ Network constructed from 64 rapidly evolving *Pm3* gene sequences from tetraploid wheats

**Fig. D** Model of the pace of genetic identity disruption after intermixing distinct populations

**References**

**Table A. List of wheat accessions**

| **Species** | **Reclassification^a^** | **Accession ID** | **Code** | **Country** | **Source^b^** | **Latitude; Longitude** |
| --- | --- | --- | --- | --- | --- | --- |
| *T.tu.* subsp. *dicoccoides* | - | IG 116188 | M001 | TUR | ICARDA | 36.867222; 36.95 |
| *T.tu.* subsp. *dicoccoides* | - | IG 116185 | M002 | TUR | ICARDA | 36.780833; 37.283333 |
| *T.tu.* subsp. *dicoccoides* | - | IG 116184 | M003 | TUR | ICARDA | 36.778333; 37.131944 |
| *T.tu.* subsp. *dicoccoides* | - | IG 46448 | M004 | SYR | ICARDA | 32.769444; 36.783333 |
| *T.tu.* subsp. *dicoccoides* | - | IG 46445 | M005 | SYR | ICARDA | 32.716667; 36.666667 |
| *T.tu.* subsp. *dicoccoides* | - | IG 46444 | M006 | SYR | ICARDA | 32.665; 36.837778 |
| *T.tu.* subsp. *dicoccoides* | - | IG 46442 | M007 | SYR | ICARDA | 32.509444; 36.808333 |
| *T.tu.* subsp. *dicoccoides* | - | IG 46439 | M008 | SYR | ICARDA | 32.490556; 36.616944 |
| *T.tu.* subsp. *dicoccoides* | - | IG 46391 | M009 | JOR | ICARDA | 32.166667; 35.916667 |
| *T.tu.* subsp. *dicoccoides* | - | IG 46384 | M010 | JOR | ICARDA | 32.3; 35.916667 |
| *T.tu.* subsp. *dicoccoides* | - | IG 110815 | M011 | LBN | ICARDA | 33.566667; 35.716667 |
| *T.tu.* subsp. *dicoccoides* | - | IG 110732 | M012 | SYR | ICARDA | 34.973056; 36.751667 |
| *T.tu.* subsp. *dicoccoides* | - | IG 109085 | M013 | IRQ | ICARDA | 36.383333; 41.783333 |
| *T.tu.* subsp. *dicoccoides* | - | IG 46527 | M014 | LBN | ICARDA | 33.433333; 35.766667 |
| *T.tu.* subsp. *dicoccoides* | - | IG 46528 | M015 | LBN | ICARDA | 34.016667; 36.083333 |
| *T.tu.* subsp. *dicoccoides* | - | IG 46525 | M016 | LBN | ICARDA | 33.516667; 35.766667 |
| *T.tu.* subsp. *dicoccoides* | - | IG 46483 | M017 | JOR | ICARDA | 32.066667; 35.75 |
| *T.tu.* subsp. *dicoccoides* | - | IG 46390 | M018 | JOR | ICARDA | 32.183333; 35.85 |
| *T.tu.* subsp. *dicoccoides* | - | IG 46472 | M019 | SYR | ICARDA | 33.644444; 35.958333 |
| *T.tu.* subsp. *dicoccoides* | - | IG 46354 | M020 | JOR | ICARDA | 32.066667; 35.75 |
| *T.tu.* subsp. *dicoccoides* | - | IG 46458 | M021 | SYR | ICARDA | 32.533333; 36.711111 |
| *T.tu.* subsp. *dicoccoides* | - | IG 117894 | M022 | SYR | ICARDA | 36.370833; 36.855 |
| *T.tu.* subsp. *dicoccoides* | - | IG 117890 | M023 | SYR | ICARDA | 36.351944; 36.85 |
| *T.tu.* subsp. *dicoccoides* | - | IG 46369 | M024 | TUR | ICARDA | 38.216667; 39.816667 |
| *T.tu.* subsp. *dicoccoides* | - | IG 113301 | M025 | IRN | ICARDA | 33.616667; 46.45 |
| *T.tu.* subsp. *dicoccoides* | - | IG 113302 | M026 | IRN | ICARDA | 33.616667; 46.45 |
| *T.tu.* subsp. *dicoccoides* | - | IG 115800 | M027 | JOR | ICARDA | 32.416667; 35.916667 |
| *T.tu.* subsp. *dicoccoides* | - | IG 115808 | M028 | JOR | ICARDA | 30.666667; 35.616667 |
| *T.tu.* subsp. *dicoccoides* | - | IG 109088 | M029 | IRQ | ICARDA | 36.35; 41.85 |
| *T.tu.* subsp. *dicoccoides* | *T.ti.* subsp*. armeniacum* | IG 46246 | M030 | TUR | ICARDA | 37.916667; 41.533333 |
| *T.tu.* subsp. *dicoccoides* | - | IG 46148 | M031 | TUR | ICARDA | 37.666667; 39.55 |
| *T.tu.* subsp. *dicoccoides* | - | IG 46248 | M032 | TUR | ICARDA | 37.933333; 40.516667 |
| *T.tu.* subsp. *dicoccoides* | *T.ti.* subsp*. armeniacum* | IG 46434 | M033 | TUR | ICARDA | 38.1; 38.516667 |
| *T.tu.* subsp. *dicoccoides* | - | IG 46244 | M034 | TUR | ICARDA | 37.716667; 39.45 |
| *T.tu.* subsp. *dicoccoides* | - | PI 428063 | M035 | TUR | NSGC | 37.8; 39.766667 |
| *T.tu.* subsp. *dicoccoides* | - | PI 428069 | M036 | TUR | NSGC | 37.866667; 39.883333 |
| *T.tu.* subsp. *dicoccoides* | - | PI 428086 | M037 | TUR | NSGC | 37.716667; 39.5 |
| *T.tu.* subsp. *dicoccoides* | - | PI 428145 | M038 | TUR | NSGC | 37.283333; 41.6 |
| *T.tu.* subsp. *dicoccoides* | - | PI 538660 | M039 | TUR | NSGC | 37.783333; 39.766667 |
| *T.tu.* subsp. *dicoccoides* | *T.ti.* subsp*. armeniacum* | PI 560872 | M040 | TUR | NSGC | 37.933333; 42.266667 |
| *T.tu.* subsp. *dicoccoides* | *T.ti.* subsp*. armeniacum* | PI 560877 | M041 | TUR | NSGC | 38.216667; 41.433333 |
| *T.tu.* subsp. *dicoccoides* | - | PI 428053 | M042 | TUR | NSGC | 37.833333; 39.816667 |
| *T.tu.* subsp. *dicoccoides* | *T.ti.* subsp*. armeniacum* | PI 560874 | M043 | TUR | NSGC | 37.733333; 42.25 |
| *T.tu.* subsp. *dicoccoides* | *T.ti.* subsp*. armeniacum* | PI 560873 | M044 | TUR | NSGC | 37.783333; 42.05 |
| *T.tu.* subsp. *dicoccoides* | *T.ti.* subsp*. armeniacum* | PI 560697 | M045 | TUR | NSGC | 37.583333; 42.383333 |
| *T.tu.* subsp. *dicoccoides* | - | PI 554583 | M046 | TUR | NSGC | 37.783333; 39.783333 |
| *T.tu.* subsp. *dicoccoides* | - | - | M047 | LBN | TB | 33.884722; 35.551389 |
| *T.tu.* subsp. *dicoccoides* | - | - | M048 | ISR | TB | 32.716667; 35.0 |
| *T.tu.* subsp. *dicoccoides* | - | - | M049 | LBN | TB | 33.884722; 35.551389 |
| *T.tu.* subsp. *dicoccoides* | - | - | M050 | LBN | TB | 33.884722; 35.551389 |
| *T.tu.* subsp. *dicoccoides* | - | TRI16627 | M051 | ISR | IPK | unavailable |
| *T.tu.* subsp. *dicoccoides* | - | TRI16625 | M052 | ISR | IPK | 33.23348; 35.78769^c^ |
| *T.tu.* subsp. *dicoccoides* | - | TRI11502 | M053 | TUR | IPK | unavailable |
| *T.tu.* subsp. *dicoccoides* | - | TRI11505 | M054 | LBN | IPK | unavailable |
| *T.tu.* subsp. *dicoccoides* | - | TRI11504 | M055 | LBN | IPK | unavailable |
| *T.tu.* subsp. *dicoccoides* | - | TRI16626 | M056 | ISR | IPK | 33.26772; 35.77318^c^ |
| *T.tu.* subsp. *dicoccoides* | - | TRI16629 | M057 | ISR | IPK | 32.9374; 35.45037^c^ |
| *T.tu.* subsp. *dicoccoides* | - | TRI16630 | M058 | ISR | IPK | unavailable |
| *T.tu.* subsp. *dicoccoides* | *T.ti.* subsp*. armeniacum* | TRI9865 | M059 | former SUN | IPK | unavailable |
| *T.tu.* subsp. *dicoccoides* | - | TRI17214 | M060 | ISR | IPK | unavailable |
| *T.tu.* subsp. *dicoccoides* | - | IG 46519 | M061 | SYR | ICARDA | 32.833611; 36.175 |
| *T.tu.* subsp. *dicoccoides* | - | TRI16633 | M062 | ISR | IPK | 33.19173; 35.61909^c^ |
| *T.tu.* subsp. *dicoccum* | - | IG 45401 | M063 | GEO | ICARDA | 42.016667; 44.933333 |
| *T.tu.* subsp. *dicoccum* | - | IG 45243 | M064 | ITA | ICARDA | 40.833333; 15.483333 |
| *T.tu.* subsp. *dicoccum* | - | IG 45247 | M065 | ITA | ICARDA | 40.65; 15.583333 |
| *T.tu.* subsp. *dicoccum* | - | IG 45338 | M066 | RUS | ICARDA | unavailable |
| *T.tu.* subsp. *dicoccum* | - | IG 45327 | M067 | RUS | ICARDA | unavailable |
| *T.tu.* subsp. *dicoccum* | - | IG 45359 | M068 | IRN | ICARDA | unavailable |
| *T.tu.* subsp. *dicoccum* | - | IG 45345 | M069 | IRN | ICARDA | unavailable |
| *T.tu.* subsp. *dicoccum* | - | IG 45308 | M070 | IND | ICARDA | unavailable |
| *T.tu.* subsp. *dicoccum* | - | IG 45334 | M071 | ARM | ICARDA | unavailable |
| *T.tu.* subsp. *dicoccum* | - | IG 45426 | M072 | HUN | ICARDA | 47.483333; 19.066667 |
| *T.tu.* subsp. *dicoccum* | - | IG 45408 | M073 | UKR | ICARDA | 49.85; 35.6 |
| *T.tu.* subsp. *dicoccum* | - | IG 45399 | M074 | ROM | ICARDA | unavailable |
| *T.tu.* subsp. *dicoccum* | - | IG 45364 | M075 | CHE | ICARDA | unavailable |
| *T.tu.* subsp. *dicoccum* | - | IG 45363 | M076 | PSE | ICARDA | unavailable |
| *T.tu.* subsp. *dicoccum* | - | IG 45410 | M077 | BLR | ICARDA | 52.78; 29.45 |
| *T.tu.* subsp. *dicoccoides* | - | IG 46386 | M078 | JOR | ICARDA | 31.85; 35.8 |
| *T. zhukovskyi* | *T.tu. s*ubsp. *dicoccum* | IG 45441 | M079 | SYR | ICARDA | unavailable |
| *T.tu.* subsp. *dicoccum* | - | IG 45406 | M080 | MNE | ICARDA | 42.766667; 18.816667 |
| *T.tu.* subsp. *dicoccum* | - | IG 45384 | M081 | SRB | ICARDA | 44.8; 20.466667 |
| *T.tu.* subsp. *dicoccum* | - | IG 45087 | M082 | TUR | ICARDA | 40.766667; 43.283333 |
| *T.tu.* subsp. *dicoccum* | - | IG 45391 | M083 | TUR | ICARDA | 40.766667; 43.283333 |
| *T.tu.* subsp. *dicoccum* | - | IG 45414 | M084 | DEU | ICARDA | unavailable |
| *T.tu.* subsp. *dicoccum* | - | IG 45350 | M085 | DEU | ICARDA | unavailable |
| *T.tu.* subsp. *dicoccum* | - | IG 45398 | M086 | ESP | ICARDA | 42.75; -1.666667 |
| *T.tu.* subsp. *dicoccum* | - | IG 45096 | M087 | ESP | ICARDA | 42.866667; -1.75 |
| *T.tu.* subsp. *dicoccum* | - | IG 45411 | M088 | MAR | ICARDA | unavailable |
| *T.tu.* subsp. *dicoccum* | - | IG 45393 | M089 | ERI | ICARDA | 15; 39 |
| *T.tu.* subsp. *dicoccum* | - | IG 45321 | M090 | ETH | ICARDA | 8.6; 39.116667 |
| *T.tu.* subsp. *dicoccum* | - | TRI11283 | M091 | SVK | IPK | unavailable |
| *T.tu.* subsp. *dicoccum* | - | TRI11293 | M092 | SVK | IPK | unavailable |
| *T.tu.* subsp. *dicoccum* | - | TRI3424 | M093 | ISR | IPK | unavailable |
| *T.ti.* subsp. *timopheevii* | - | IG 46247 | M094 | TUR | ICARDA | 38.183333; 41.516667 |
| *T.tu.* subsp. *turgidum* | - | - | M095 | GBR | TB | unavailable |
| *T.tu.* subsp. *dicoccoides* | - | IG 131234 | M096 | IRN | ICARDA | 34.783333; 46.933333 |
| *T.tu.* subsp. *dicoccoides* | - | IG 116173 | M097 | TUR | ICARDA | 36.705556; 37.230278 |
| *T.tu.* subsp. *dicoccoides* | - | IG 46475 | M098 | SYR | ICARDA | 33.812778; 36.163889 |
| *T.tu.* subsp. *dicoccoides* | - | IG 46521 | M099 | SYR | ICARDA | 33.013889; 36.041667 |
| *T.tu.* subsp. *dicoccoides* | - | IG 46477 | M100 | SYR | ICARDA | 33.790278; 36.094444 |
| *T.tu.* subsp. *dicoccoides* | - | IG 46506 | M101 | SYR | ICARDA | 36.475; 36.991667 |
| *T.tu.* subsp. *dicoccoides* | - | IG 116181 | M102 | TUR | ICARDA | 37.322778; 37.467222 |
| *T.tu.* subsp. *dicoccoides* | *T.ti.* subsp*. armeniacum* | IG 116176 | M103 | TUR | ICARDA | 37.270833; 37.540278 |
| *T.tu.* subsp. *dicoccoides* | - | IG 116179 | M104 | TUR | ICARDA | 37.253056; 37.333056 |
| *T.tu.* subsp. *dicoccum* | - | IG 45444 | M105 | PSE | ICARDA | unavailable |
| *T.tu.* subsp. *dicoccum* | - | IG 45352 | M108 | CHN (?) | ICARDA | 45.733333; 126.6 |
| *T.tu.* subsp. *dicoccum* | - | IG 4318 | M109 | AFG | ICARDA | unavailable |
| *T.tu.* subsp. *dicoccum* | - | IG 45073 | M110 | OMN | ICARDA | 22.166667; 59.166667 |
| *T.tu.* subsp. *dicoccum* | - | IG 45070 | M111 | OMN | ICARDA | 23.416111; 57.133333 |
| *T.tu.* subsp. *dicoccum* | - | IG 45305 | M112 | IND | ICARDA | 17.33; 77.9 |
| *T.tu.* subsp. *dicoccum* | - | IG 45254 | M113 | JOR | ICARDA | 32.066667; 35.75 |
| *T.tu.* subsp. *dicoccum* | - | IG 45392 | M114 | TUR | ICARDA | 40.766667; 43.283333 |
| *T.tu.* subsp. *dicoccum* | - | IG 45336 | M115 | TUR | ICARDA | 40.766667; 43.283333 |
| *T.tu.* subsp. *dicoccum* | - | IG 45335 | M116 | TUR | ICARDA | 40.766667; 43.283333 |
| *T.tu.* subsp. *dicoccum* | - | IG 45089 | M117 | TUR | ICARDA | 40.766667; 43.283333 |
| *T.tu.* subsp. *dicoccum* | - | IG 45088 | M118 | TUR | ICARDA | 40.766667; 43.283333 |
| *T.tu.* subsp. *dicoccum* | - | IG 45087 | M119 | TUR | ICARDA | 40.766667; 43.283333 |
| *T.tu.* subsp. *dicoccum* | - | IG 45391 | M120 | TUR | ICARDA | 40.766667; 43.283333 |
| *T.tu.* subsp. *dicoccum* | - | IG 45412 | M121 | IRN | ICARDA | unavailable |
| *T.tu.* subsp. *dicoccum* | - | IG 45400 | M122 | IRN | ICARDA | unavailable |
| *T.tu.* subsp. *dicoccum* | - | IG 45330 | M123 | IRN | ICARDA | unavailable |
| *T.tu.* subsp. *dicoccum* | - | IG 45331 | M124 | IRN | ICARDA | unavailable |
| *T.tu.* subsp. *dicoccum* | - | IG 45341 | M125 | IRN | ICARDA | unavailable |
| *T.tu.* subsp. *dicoccum* | - | IG 45342 | M126 | IRN | ICARDA | unavailable |
| *T.tu.* subsp. *dicoccum* | - | IG 45343 | M127 | IRN | ICARDA | unavailable |
| *T.tu.* subsp. *dicoccum* | - | IG 45344 | M128 | IRN | ICARDA | unavailable |
| *T.tu.* subsp. *dicoccum* | - | IG 45346 | M129 | IRN | ICARDA | unavailable |
| *T.tu.* subsp. *dicoccum* | - | IG 45347 | M130 | IRN | ICARDA | unavailable |
| *T.tu.* subsp. *dicoccum* | - | IG 45351 | M131 | IRN | ICARDA | unavailable |
| *T.tu.* subsp. *dicoccum* | - | IG 45381 | M132 | IRN | ICARDA | unavailable |
| *T.tu.* subsp. *dicoccum* | - | IG 45382 | M133 | IRN | ICARDA | unavailable |
| *T.tu.* subsp. *dicoccum* | - | IG 45396 | M134 | IRN | ICARDA | unavailable |
| *T.tu.* subsp. *dicoccum* | - | IG 45340 | M135 | IRN | ICARDA | unavailable |
| *T.tu.* subsp. *dicoccum* | - | TRI584 | M136 | TUR | IPK | unavailable |
| *T.tu.* subsp. *dicoccum* | - | TRI17040 | M137 | TUR | IPK | unavailable |
| *T.tu.* subsp. *dicoccum* | - | TRI17031 | M138 | TUR | IPK | 40.75; 43.416667 |
| *T.tu.* subsp. *dicoccum* | - | TRI17029 | M139 | TUR | IPK | 41.35; 33.85 |
| *T.tu.* subsp. *dicoccum* | - | TRI6158 | M140 | IRN | IPK | 32.55; 50.85 |
| *T.tu.* subsp. *dicoccum* | - | TRI6141 | M141 | IRN | IPK | 32.55; 50.85 |
| *T.tu.* subsp. *dicoccum* | - | TRI6142 | M142 | IRN | IPK | 32.55; 50.85 |
| *T.tu.* subsp. *dicoccum* | - | TRI3432 | M143 | KWT | IPK | unavailable |
| *T.ti.* subsp*. armeniacum* | - | CItr17678 | S001 | ARM | NSGC | unavailable |
| *T.ti.* subsp*. armeniacum* | - | PI 355452 | S002 | AZE | NSGC | 40.5; 45 |
| *T.ti.* subsp*. armeniacum* | - | AZESVK-47 | S003 | AZE | RIPP | unavailable |
| *T.ti.* subsp*. armeniacum* | - | PI 427366 | S004 | IRN | NSGC | 34.3; 46.183333 |
| *T.ti.* subsp*. armeniacum* | - | PI 427347 | S005 | IRQ | NSGC | 36.916667; 43.033333 |
| *T.ti.* subsp*. armeniacum* | - | CItr17677 | S006 | TUR | NSGC | 38.983333; 39.5 |
| *T.ti.* subsp*. armeniacum* | *T.tu.* subsp. *dicoccoides* | PI 427998 | S007 | LEB | NSGC | 33.516667; 35.866667 |
| *T.ti.* subsp*. timopheevii* | - | PI 352506 | S008 | GEO | NSGC | unavailable |
| *T.ti.* subsp*. timopheevii* | - | PI 352508 | S009 | GEO | NSGC | unavailable |
| *T.ti.* subsp*. timopheevii* | *T.tu.* subsp. *dicoccum* | PI 251017 | S010 | RUS | NSGC | unavailable |
| *T.ti.* subsp*. timopheevii* | *T.tu.* subsp. *dicoccum* | PI 251018 | S011 | UZB | NSGC | 41.333333; 69.3 |
| *T.ti.* subsp*. timopheevii* | - | PI 119442 | S012 | TUR | NSGC | 41.242222; 33.328333 |
| *T.ti.* subsp*. timopheevii* | - | CItr15205 | S013 | GRC | NSGC | unavailable |
| *T.ti.* subsp*. timopheevii* | - | PI 221421 | S014 | SRB | NSGC | unavailable |
| *T.tu.* subsp. *dicoccoides* | - | PI 352322 | S015 | LBN | NSGC | 33.416667; 35.866667 |
| *T.tu.* subsp. *dicoccoides* | - | IG 131233 | S016 | IRQ | ICARDA | 37.1; 43.633333 |
| *T.tu.* subsp. *dicoccoides* | - | IG 131232 | S017 | IRQ | ICARDA | 36.6; 44.486389 |
| *T.tu.* subsp. *dicoccoides* | *T.ti.* subsp*. armeniacum* | IG 131231 | S018 | IRQ | ICARDA | 35.4; 45.5 |
| *T.tu.* subsp. *dicoccoides* | - | PI 554580 | S019 | TUR | NSGC | 37.783333; 39.766667 |
| *T.tu.* subsp. *dicoccoides* | - | PI 428016 | S020 | IRN | NSGC | 34.366667; 46.1 |
| *T.tu.* subsp. *dicoccoides* | - | 01C0201521 | S021 | ISR | RICP | unavailable |
| *T. ispahanicum* | - | PI 352492 | S022 | IRN | NSGC | unavailable |
| *T. ispahanicum* | - | PI 352493 | S023 | IRN | NSGC | unavailable |
| *T. ispahanicum* | - | PI 572904 | S024 | IRN | NSGC | unavailable |
| *T. ispahanicum* | - | PI 294477 | S025 | IRN | NSGC | unavailable |
| *T. ispahanicum* | - | PI 294478 | S026 | IRN | NSGC | unavailable |
| *T. ispahanicum* | - | PI 330548 | S027 | IRN | NSGC | unavailable |
| *T. ispahanicum* | - | PI 346782 | S028 | HUN (?) | NSGC | 47.416667; 19.333333 |
| *T. ispahanicum* | - | 01C0201721 | S029 | IRN | RICP | unavailable |
| *T. ispahanicum* | - | AZESVK2009-92 | S030 | ARM (?) | RIPP | unavailable |
| *T.tu.* subsp. *carthlicum* | - | PI 61102 | S031 | GEO | NSGC | unavailable |
| *T.tu.* subsp. *carthlicum* | - | PI 283887 | S032 | IRN | NSGC | unavailable |
| *T.tu.* subsp. *carthlicum* | - | PI 283888 | S033 | IRN | NSGC | unavailable |
| *T.tu.* subsp. *carthlicum* | - | PI 283889 | S034 | IRN | NSGC | unavailable |
| *T.tu.* subsp. *carthlicum* | - | PI 387696 | S035 | ETH | NSGC | unavailable |
| *T.tu.* subsp. *carthlicum* | - | PI 573179 | S036 | TUR | NSGC | 40.783333; 43.016667 |
| *T.tu.* subsp. *carthlicum* | - | PI 573182 | S037 | TUR | NSGC | 40.95; 43.283333 |
| *T.tu.* subsp. *polonicum* | - | PI 167622 | S038 | TUR | NSGC | 39.913611; 28.157778 |
| *T.tu.* subsp. *polonicum* | - | PI 208911 | S039 | IRQ | NSGC | unavailable |
| *T.tu.* subsp. *polonicum* | - | PI 223171 | S040 | JOR | NSGC | unavailable |
| *T.tu.* subsp. *polonicum* | - | PI 225335 | S041 | IRN | NSGC | unavailable |
| *T.tu.* subsp. *polonicum* | - | PI 245663 | S042 | AFG | NSGC | unavailable |
| *T.tu.* subsp. *polonicum* | - | PI 254215 | S043 | IRQ | NSGC | unavailable |
| *T.tu.* subsp. *polonicum* | - | 01C0201287 | S044 | TUR | RICP | unavailable |
| *T.tu.* subsp. *durum* | - | PI 182717 | S045 | SYR | NSGC | 36.5; 41 |
| *T.tu.* subsp. *turanicum* | - | PI 481582 | S046 | IRQ | NSGC | unavailable |
| *T.tu.* subsp. *turanicum* | - | PI 560896 | S047 | TUR | NSGC | 38.266667; 41.4 |
| *T.tu.* subsp. *turanicum* | - | PI 624207 | S048 | IRN | NSGC | 34.316667; 47.066667 |
| *T.tu.* subsp. *turanicum* | - | PI 625214 | S049 | IRN | NSGC | 36.666667; 48.5 |
| *T.tu.* subsp. *turanicum* | - | BVAL-212017 | S050 | unavailable | AGES | unavailable |
| *T.tu.* subsp. *turanicum* | - | KAMUT® | S051 | EGY | RIPP | unavailable |
| *T.tu.* subsp. *durum* | - | PI 61114 | S052 | IRN | NSGC | unavailable |
| *T.tu.* subsp. *durum* | - | 39-317 | S053 | ISR | IGB | unavailable |
| *T.tu.* subsp. *durum* | - | 39-321 | S054 | ISR | IGB | unavailable |
| *T.tu.* subsp. *durum* | - | 56-634 | S055 | ISR | IGB | unavailable |
| *T.tu.* subsp. *durum* | - | AZESVK2009-92 | S056 | AZE (?) | RIPP | unavailable |
| *T.tu.* subsp. *durum* | - | 01C0200668 | S057 | USA | RICP | unavailable |
| *T.tu.* subsp. *durum* | - | 01C0202874 | S058 | ITA | RICP | unavailable |
| *T.tu.* subsp. *turgidum* | - | PI 66058 | S059 | EGY | NSGC | 30.033333; 31.216667 |
| *T.tu.* subsp. *turgidum* | - | PI 223173 | S060 | JOR | NSGC | unavailable |
| *T.tu.* subsp. *turgidum* | - | PI 341482 | S061 | TUR | NSGC | 38.079444; 31.366389 |
| *T.tu.* subsp. *turgidum* | - | NGB 4795 | S062 | DNK | NGB | unavailable |
| *T.tu.* subsp. *turgidum* | - | NGB 9012 | S063 | DNK | NGB | unavailable |
| *T.tu.* subsp. *turgidum* | - | NGB 9050 | S064 | DNK | NGB | unavailable |
| *T.tu.* subsp. *turgidum* | - | 22490 | S065 | PRT | IHAR | unavailable |
| *T.tu.* subsp. *dicoccum* | - | TRI17023 | S068 | TUR | IPK | 41.683333; 33.716667 |
| *T.tu.* subsp. *dicoccum* | - | PI 319869 | S069 | TUR | NSGC | unavailable |
| *T.tu.* subsp. *dicoccum* | - | PI 606325 | S070 | TUR | NSGC | 41.415833; 35.054444 |
| *T.tu.* subsp. *dicoccum* | - | K-23035 | S071 | former YUG | VIR | unavailable |
| *T.tu.* subsp. *dicoccum* | - | K-35099 | S072 | RUS | VIR | unavailable |
| *T.tu.* subsp. *dicoccum* | - | PI 168676 | S073 | ETH | NSGC | unavailable |
| *T.tu.* subsp. *dicoccum* | - | CItr14868 | S074 | ETH | NSGC | 10.35; 37.733333 |
| *T.tu.* subsp. *dicoccum* | - | PI 191387 | S075 | ETH | NSGC | 9.5; 41.5 |
| *T.tu.* subsp. *dicoccum* | - | K-5154 | S076 | ETH | VIR | unavailable |
| *T.tu.* subsp. *dicoccum* | - | K-5202 | S077 | ETH | VIR | unavailable |
| *T.tu.* subsp. *dicoccum* | - | K-15840 | S078 | MAR | VIR | unavailable |
| *T.tu.* subsp. *dicoccum* | - | K-22246 | S079 | MAR | VIR | unavailable |
| *T.tu.* subsp. *dicoccum* | - | PI 182743 | S080 | TUR | NSGC | 37.363333; 40.271111 |
| *T.tu.* subsp. *dicoccum* | - | 01C0204035 | S081 | PSE | RICP | unavailable |
| *T.tu.* subsp. *dicoccum* | - | CGN8354 | S082 | ISR | CGN | unavailable |
| *T.tu.* subsp. *dicoccum* | - | PI 355495 | S083 | PSE | NSGC | unavailable |
| *T.tu.* subsp. *dicoccum* | - | PI 355464 | S084 | unavailable | NSGC | unavailable |
| *T.tu.* subsp. *dicoccum* | - | NGB16080 | S085 | DNK/SWE | NGB | unavailable |
| *T.tu.* subsp. *dicoccum* | - | TRI 17700 | S086 | ESP | IPK | unavailable |
| *T.tu.* subsp. *dicoccum* | - | CGN11488 | S087 | DEU | CGN | unavailable |
| *T.tu.* subsp. *paleocolchicum* | - | PI 330553 | S088 | unavailable | NSGC | unavailable |

^a^ Fourteen accessions were reclassified prior to the work described in this paper. Ten of these were accessions labelled as *T. turgidum* subsp. *dicoccoides* which we reclassified as *T. timopheevii* subsp. *armeniacum*, one was subsp. *armeniacum* reclassified as subsp. *dicoccoides*, and two *T. timopheevii* subsp. *timopheevii* reclassified as *T. turgidum* subsp. *dicoccum*. Difficulties in distinguishing BA^u^ and GA^u^ wheats have previously been documented [1.2]. The ten *dicoccoides* accessions (wild BA^u^) reclassified as *armeniacum* (wild GA^u^) were all from the natural *armeniacum* range (Mori et al. 2009) including areas in which *dicoccoides* has never been recorded, as far as we are aware. The single *armeniacum* to *dicoccoides* reclassification was an accession from Lebanon, where no *armeniacum* populations are known. The two *timopheevii* (domesticated GA^u^) to *dicoccum* (domesticated BA^u^) reclassifications were accessions from Russia and Uzbekistan, outside of the range for domesticated *timopheevii* wheat, which is limited to Georgia [2]. Additionally, an accession labelled *T. zhukovskyi* (domesticated GA^u^A^u^ hexaploid) by ICARDA, from whom we obtained seed, but described by NSGC as *T. dicoccon* (syn. *T. turgidum* subsp. *dicoccum*), was considered more likely to be the latter. All but two of these reclassifications were supported by sequence data for the *SBEIIa*-intron9, *11B* gene and *psi-LPX-A1* pseudogene (Civáň P and Ivaničová Z, unpublished data). The two accessions not tested in this way are IG 116176 and IG 131231. Neither of these two accessions is critical to any of the conclusions drawn in this paper.

^b^ Abbreviations: AGES, Österreichische Agentur für Gesundheit und Ernährungssicherheit, Linz, Austria; CGN, Centre for Genetic Resources, Wageningen, Netherlands; ICARDA, International Center for Agricultural Research in Dry Areas, Aleppo, Syria; IGB, The Israeli Gene Bank for Agricultural Crops, Bet Dagan, Israel; NGB, Nordic Genetic Resource Center, Alnarp, Sweden; NSGC, National Small Grains Collection, Aberdeen, Idaho; RICP, Research Institute of Crop Production, Prague, Czech Republic; RIPP, Research Institute of Plant Production, Piešťany, Slovakia; TB, collection of T.A.Brown; VIR, N.I.Vavilov Research Institute of Plant Industry, Petersburg, Russia.

^c^ Estimated according to given geographic description.**Table B. Primer sequences**

| **Target sequence** | **Primer** | **5'→3' sequence** |
| --- | --- | --- |
| 5S rDNA  5.8S rDNA | 5S_Id^a^ | 6FAM-AAGTCCTCGTGTTGCATTCC |
|  | 5S_Ii^a^ | 6FAM-GGAATGCAACACGAGGACTT |
|  | 5S_IId^a^ | 6FAM-TCATACCAGCACTAAAGCACC |
|  | 5S_IIi^a^ | 6FAM-GGTGCTTTAGTGCTGGTATGA |
|  | 5.8S_i^c^ | CAACTTGCGTTCAAAGACTCG |
| LTRs | *Fatima*-LTR | GTTTTACCTCCATCAAGAGGGC |
|  | *Laura*-LTR | TGAGGCCGCAAAGTGATATG |
|  | *Sabrina*-LTR | AGAGAAGCCACTAGTGAAACCT |
|  | *Wham*-LTR | AACGGACTCTAGACGAGCC |
|  | *Erika*-LTR | GAAACTTGACAGGCGGTCTAC |
|  | *Daniela*-LTR | GGAGTAGGGTTTTACGCATCC |
|  | *BARE1/Wis/Angela*-LTR^b^ | ATTGCCTCTAGGGCATATTTCC |
|  | *Jeli*-LTR^b^ | CTGTCAGACTTAGAACCACGAC |
|  | *Angela*-LTR^b^ | GCCTCTAGGGCATATTTCCTT |

^a,b^ Combining primers "a" (primers P2, P3, P4 and P5 in Figure 1) with primers "b" (primer P1 in Figure 1) results in size polymorphic PCR products; primer combinations utilised to screen the whole sample set were: *5S_Id–Jeli, 5S_Ii–Jeli, 5S_IId–Jeli, 5S_IIi–Jeli, 5S_Ii–Angela, 5S_IIi–Angela*.

^c^ PCRs with primer 5.8S_i and *Jeli*-LTR were in a volume of 50 µl with 1.6 mM MgCl_2_ and an annealing temperature of 59°C to increase specificity and facilitate direct sequencing of the amplicon.

**Table C. List of wheat accessions reanalyzed from data of Haudry et al. [3]**

| **Individual** | **Code^a^** | **Species** | **Accession Id.** | **Source^b^** | **Geographic Region** |
| --- | --- | --- | --- | --- | --- |
| DC10 |  | *T.tu. subsp*. *dicoccum* | 45383 | Icarda | Bulgaria |
| DC11 |  | *T.tu. subsp*. *dicoccum* | 45351 | Icarda | Iran |
| DC12 |  | *T.tu. subsp*. *dicoccum* | 45239 | Icarda | Italy |
| DC13 |  | *T.tu. subsp*. *dicoccum* | 45354 | Icarda | Russia |
| DC14 |  | *T.tu. subsp*. *dicoccum* | 45280 | Icarda | Slovakia |
| DC15 |  | *T.tu. subsp*. *dicoccum* | 45309 | Icarda | Slovakia |
| DC16 |  | *T.tu. subsp*. *dicoccum* | PI 352365 | UsDA | Germany |
| DC17 |  | *T.tu. subsp*. *dicoccum* | PI 355484 | UsDA | Spain |
| DC18 |  | *T.tu. subsp*. *dicoccum* | PI 94635 | UsDA | Iran |
| DC19 |  | *T.tu. subsp*. *dicoccum* | PI 415152 | UsDA | Israel |
| DC20 |  | *T.tu. subsp*. *dicoccum* | PI 94648 | UsDA | Italy |
| DC70 |  | *T.tu. subsp*. *dicoccum* | erge 4098 | CRB Clermont | India |
| DD21 | **SE1** | *T.tu. subsp*. *dicoccoides* | 113302 | Icarda | Iran Ilam |
| DD22 | **SW1** | *T.tu. subsp*. *dicoccoides* | 45963 | Icarda | Jordan, Amman |
| DD23 | **SW2** | *T.tu. subsp*. *dicoccoides* | 46391 | Icarda | Jordan, Zarqa |
| DD25 | **SW3** | *T.tu. subsp*. *dicoccoides* | 46470 | Icarda | Syria, As Suwayda |
| DD26 | **NE1** | *T.tu. subsp*. *dicoccoides* | 46253 | Icarda | Turkey, Diyarbakir (Karaca Dağ vicinity) |
| DD27 | **NW1** | *T.tu. subsp*. *dicoccoides* | 116172 | Icarda | Turkey, Gaziantep |
| DD28 | **SW4** | *T.tu. subsp*. *dicoccoides* | PI 467014 | UsDA | Israel |
| DD29 | **SW5** | *T.tu. subsp*. *dicoccoides* | PI 428133 | UsDA | Lebanon |
| DD30 | **SW6** | *T.tu. subsp*. *dicoccoides* | 46516 | Icarda | Syria, As Suwayda |
| DD31 | **SW7** | *T.tu. subsp*. *dicoccoides* | PI 487255 | UsDA | Syria |
| DD48 | **SW8** | *T.tu. subsp*. *dicoccoides* | 46499 | Icarda | Jordan, As Salt |
| DD51 | **SW9** | *T.tu. subsp*. *dicoccoides* | 111002 | Icarda | Jordan, Irbid |
| DD53 | **SW10** | *T.tu. subsp*. *dicoccoides* | 115811 | Icarda | Jordan, Tafila |
| DD56 | **SW11** | *T.tu. subsp*. *dicoccoides* | 46056 | Icarda | Jordan, Tafila |
| DD61 | **SW12** | *T.tu. subsp*. *dicoccoides* | 46294 | Icarda | Palestinian Territories |
| DD63 | **SW13** | *T.tu. subsp*. *dicoccoides* | 46310 | Icarda | Palestinian Territories |
| DD64 | **SW14** | *T.tu. subsp*. *dicoccoides* | 46518 | Icarda | Syria, Dar'a |
| DD65 | **SW15** | *T.tu. subsp*. *dicoccoides* | 46520 | Icarda | Syria, Dar'a |
| DD70 | **SW16** | *T.tu. subsp*. *dicoccoides* | 46501 | Icarda | Syria, As Suwayda |
| DD74 | **NW2** | *T.tu. subsp*. *dicoccoides* | 116175 | Icarda | Turkey, Gaziantep |
| DD75 | **NW3** | *T.tu. subsp*. *dicoccoides* | 116179 | Icarda | Turkey, Gaziantep |
| DD76 | **NW4** | *T.tu. subsp*. *dicoccoides* | 116184 | Icarda | Turkey, Gaziantep |
| DD77 | **NE2** | *T.tu. subsp*. *dicoccoides* | 46191 | Icarda | Turkey, Urfa (Karaca Dağ vicinity) |
| DD78 | **NE3** | *T.tu. subsp*. *dicoccoides* | 46244 | Icarda | Turkey, Urfa (Karaca Dağ vicinity) |
| DD81 | **N/A** | *T.tu. subsp*. *dicoccoides* | 68263 | rennes | Unknown |
| DD82 | **SW17** | *T.tu. subsp*. *dicoccoides* | PI 428105 | UsDA | Israel |
| DD95 | **NW5** | *T.tu. subsp*. *dicoccoides* | 117894 | Icarda | Syria, Aleppo |
| DD101 | **NE4** | *T.tu. subsp*. *dicoccoides* | PI 538657 | UsDA | Turkey, Diyarbakir (Karaca Dağ vicinity) |
| DR36 |  | *T.tu. subsp*. *durum* | senatoreCappelli(21) | Montpellier | Italy |
| DR47 |  | *T.tu. subsp*. *durum* | LRB6R5546/49/4 |  | Bulgaria |
| DR49 |  | *T.tu. subsp*. *durum* | kubanka | Montpellier | Russia |
| DR50 |  | *T.tu. subsp*. *durum* | 84866 | Icarda | Syria |
| DR51 |  | *T.tu. subsp*. *durum* | 95920 | Icarda | Syria |
| DR52 |  | *T.tu. subsp*. *durum* | 82697 | Icarda | Turkey |
| DR53 |  | *T.tu. subsp*. *durum* | 82726 | Icarda | Turkey |
| DR54 |  | *T.tu. subsp*. *durum* | 82715 | Icarda | Turkey |
| DR55 |  | *T.tu. subsp*. *durum* | BRUMAIRE | GEVEs | France |
| DR56 |  | *T.tu. subsp*. *durum* | PRIMADUR | GEVEs | France |
| DR57 |  | *T.tu. subsp*. *durum* | NEODUR91 | GEVEs | France |
| DR58 |  | *T.tu. subsp*. *durum* | IXOs9442 | GEVEs | France |
| DR59 |  | *T.tu. subsp*. *durum* | VILLEMUR | GEVEs | France |
| DR60 |  | *T.tu. subsp*. *durum* | ARMET910 | GEVEs | France |
| DR61 |  | *T.tu. subsp*. *durum* | DURENTAL | GEVEs | France |
| DR62 |  | *T.tu. subsp*. *durum* | LLOYD945 | GEVEs | France |
| DR63 |  | *T.tu. subsp*. *durum* | B6Rtchir |  | Bulgaria |
| DR75 |  | *T.tu. subsp*. *durum* | erge 3173 | CRB Clermont | Italy |
| DR77 |  | *T.tu. subsp*. *durum* | erge 7657 | CRB Clermont | Spain |
| DR78 |  | *T.tu. subsp*. *durum* | erge 3509 | CRB Clermont | Turkey |
| TI103 |  | *T. timopheevii* | PI 560872 | UsDA | Turkey |
| TI104 |  | *T. timopheevii* | PI 560873 | UsDA | Turkey |
| TI105 |  | *T. timopheevii* | PI 560874 | UsDA | Turkey |
| TI106 |  | *T. timopheevii* | PI 560877 | UsDA | Turkey |
| TI32 |  | *T. timopheevii* | PI 560697 | UsDA | Turkey |

**^a^** Geographical coding used in Figure 4.

^b^ As described but not defined by Haudry et al. [3].

^c^ The geographical origin that we give for this accession corresponds to the data given by the germplasm collection, but is different to that stated by Haudry et al. [3].

**Table D.** **Data obtained by reanalysis of sequence data for 21 loci in wild and domesticated emmer**

| **Gene** | **π×10^-3^** | **#DD** | **#DC** | **A^DC^** | **A^DC∩DD^** |
| --- | --- | --- | --- | --- | --- |
| *11B* | 3.28 | 10 | 12 | 2 | 1 |
| *91A* | 2.18 | 10 | 12 | 2 | 1 |
| *AapA* | 0.6 | 9 | 10 | 1 | 1 |
| *AlperA* | 2.28 | 23 | 12 | 3 | 3 |
| *Bp2A* | 1.67 | 27 | 12 | 3 | 2 |
| *Bp3B* | 1.04 | 10 | 8 | 2 | 1 |
| *Bp5A* | 0.83 | 26 | 12 | 1 | 1 |
| *ChsA* | 11.27 | 6 | 6 | 2 | 2 |
| *GdhA* | 2.4 | 26 | 12 | 3 | 3 |
| *Gsp1A* | 4.2 | 25 | 11 | 4 | 2 |
| *Gsp1B* | 2.23 | 28 | 12 | 5 | 2 |
| *HgA* | 11.63 | 15 | 6 | 3 | 1 |
| *HiplA* | 1.8 | 26 | 12 | 2 | 1 |
| *Mdh4B* | 1.72 | 8 | 12 | 3 | 2 |
| *MdhA* | 1.16 | 27 | 11 | 1 | 1 |
| *Mp7A* | 0.97 | 24 | 12 | 3 | 2 |
| *MybA* | 0.61 | 10 | 12 | 2 | 2 |
| *MybB* | 1.37 | 10 | 11 | 6 | 2 |
| *NrpA* | 2.58 | 18 | 9 | 3 | 2 |
| *PsyA* | 1.84 | 28 | 12 | 3 | 2 |
| *ZdsB* | 0.82 | 28 | 12 | 3 | 2 |

NOTE—*π×10^-3^*, average number of nucleotide differences per site between allele pairs [3]; *#DD*, number of sequences available for wild emmer; *#DC* number of sequences available for domesticated emmer; *A^DC^*, number of alleles detected in domesticated emmer; *A^DC∩DD^*, number of alleles shared by domesticated emmer and wild accessions. The difference between *A^DC^* and *A^DC∩DD^* may be due to post-domestication divergence or poor sampling of wild emmer.

**Fig. A.** Geographical locations of wild accessions for which *Jeli*–5.8S sequence data were obtained. (*A*) Wild emmers with alleles from clusters I and II in the MJ network (Figure 2). (*B*) Wild emmers from clusters III and IV. (*C*) Accessions with early-diverging or basal alleles. Color coding corresponds with the outer circles of the nodes in Figure 2. The locations of Bingöl (B) and Kapadokya (K) are indicated (see Discussion). In (*C*) the early-diverging and basal types of *T. turgidum* subsp. *dicoccoides* are confined largely to the western Fertile Crescent and the wild GA^u^ species *Triticum timopheevii* subsp. *armeniacum* to the eastern arm. These distributions are consistent with the presumed origin of BA^u^ tetraploids in the vicinity of Mount Hermon [4] and the reported distribution of wild *timopheevii* wheat [5].


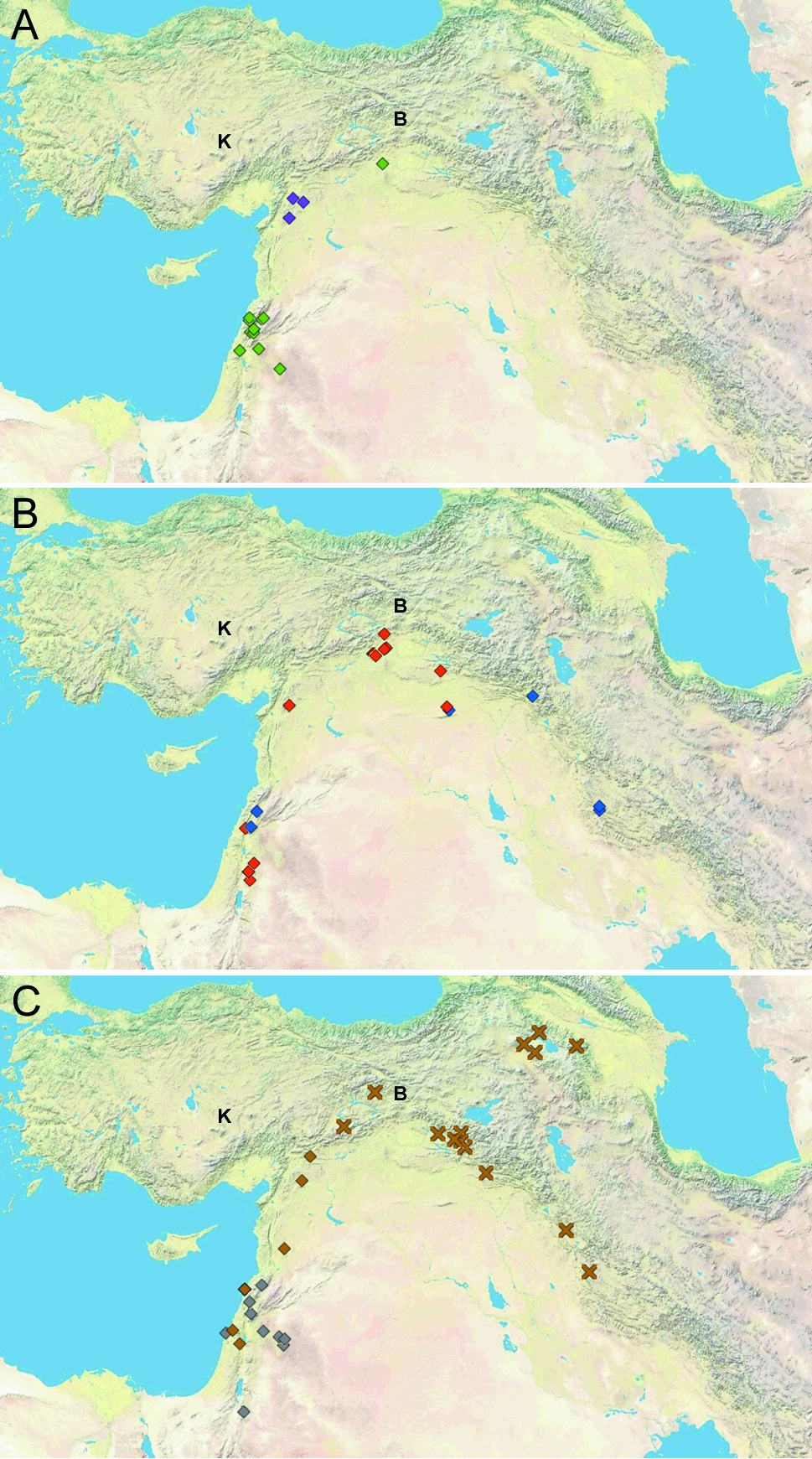


**Fig. B.** The most parsimonious tree constructed from the 21-gene concatenated data matrix. Color coding of the leafs corresponds to that used in Figure 5. Clades that obtained >50% support in the bootstrap analysis are indicated. With regards to the origin of domesticated emmer, the most frequent grouping in the bootstrap analysis involves all the *dicoccum* and *durum* accessions together with the wild samples SE1 and SW17, this group appearing in 16.8% of the resampled trees. Alternative frequent groupings exclude the domesticated sample DC19 from the set above (9.8% of the resampled trees), or add the samples NE4 (5.6%), NE4+NW2+NW3±SW10 (5.6%), NE4+SW10 (3.7%), or NE4+SW10+NE2+NE3 (3.1%).


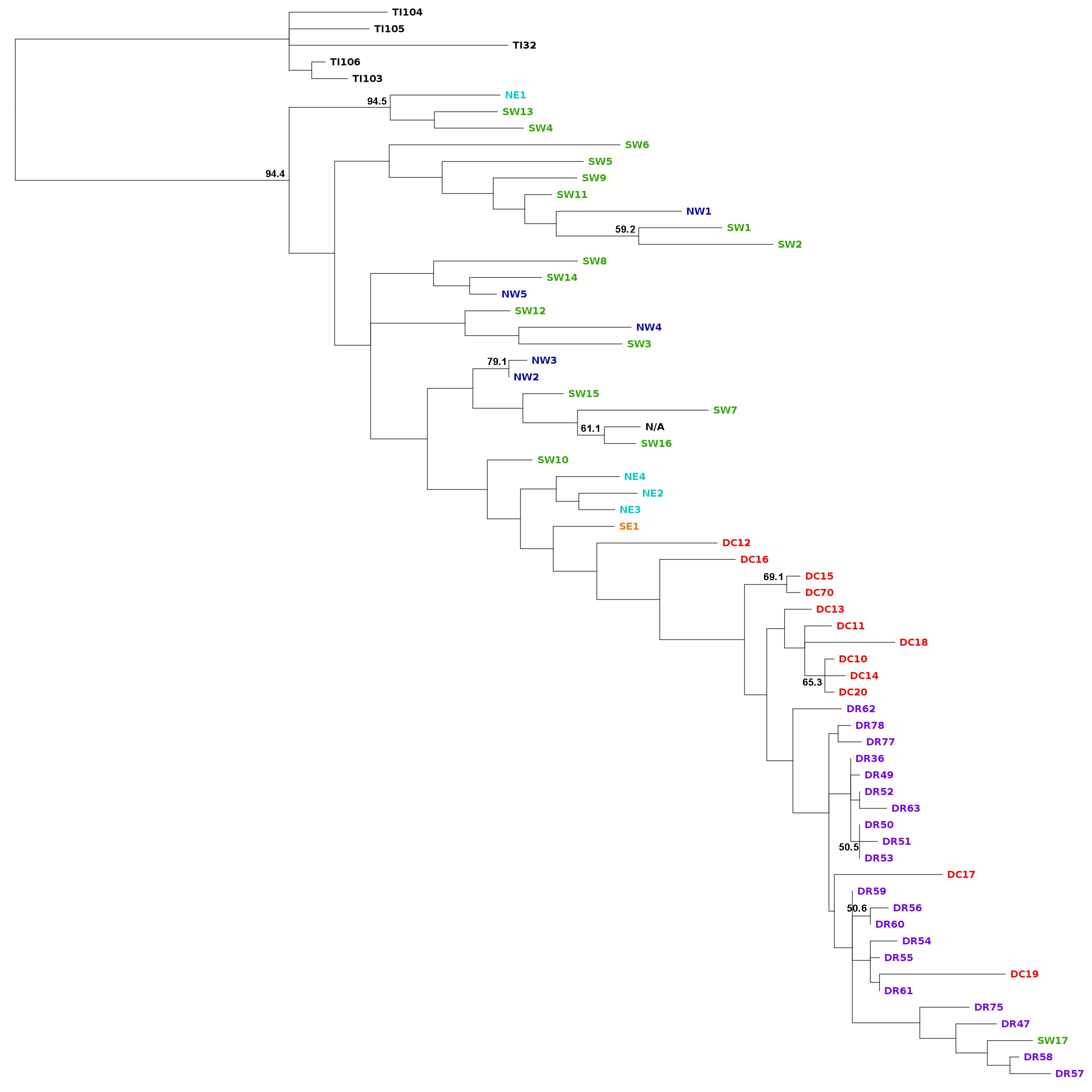


**Fig. C.** MJ Network constructed from 64 rapidly evolving *Pm3* gene sequences from tetraploid wheats [6], plus additional sequences from Genbank (excluding three that appear to display intragenic recombination). Node sizes are proportional to the number of accessions displaying that allele, and the edge lengths are proportional to the number of substitutions between pairs of allele sequences. Southwest populations originate from Israel, Lebanon and southwest Syria, northeast populations from the vicinity of Karaca Dağ Mountain, and the southeast population (a single accession) is from the Iranian Zagros Mountains. A presumed ortholog sequence from *T. monococcum* is included as outgroup. Accessions from the northeast and southeast are located in two peripheral regions of the network. The genetic distance between these two peripheral groups, and the presence of closely-related Levantine accessions at positions basal to both groups, suggest that one or both of the northeast and southeast allele groups migrated from the southern Levant relatively recently.


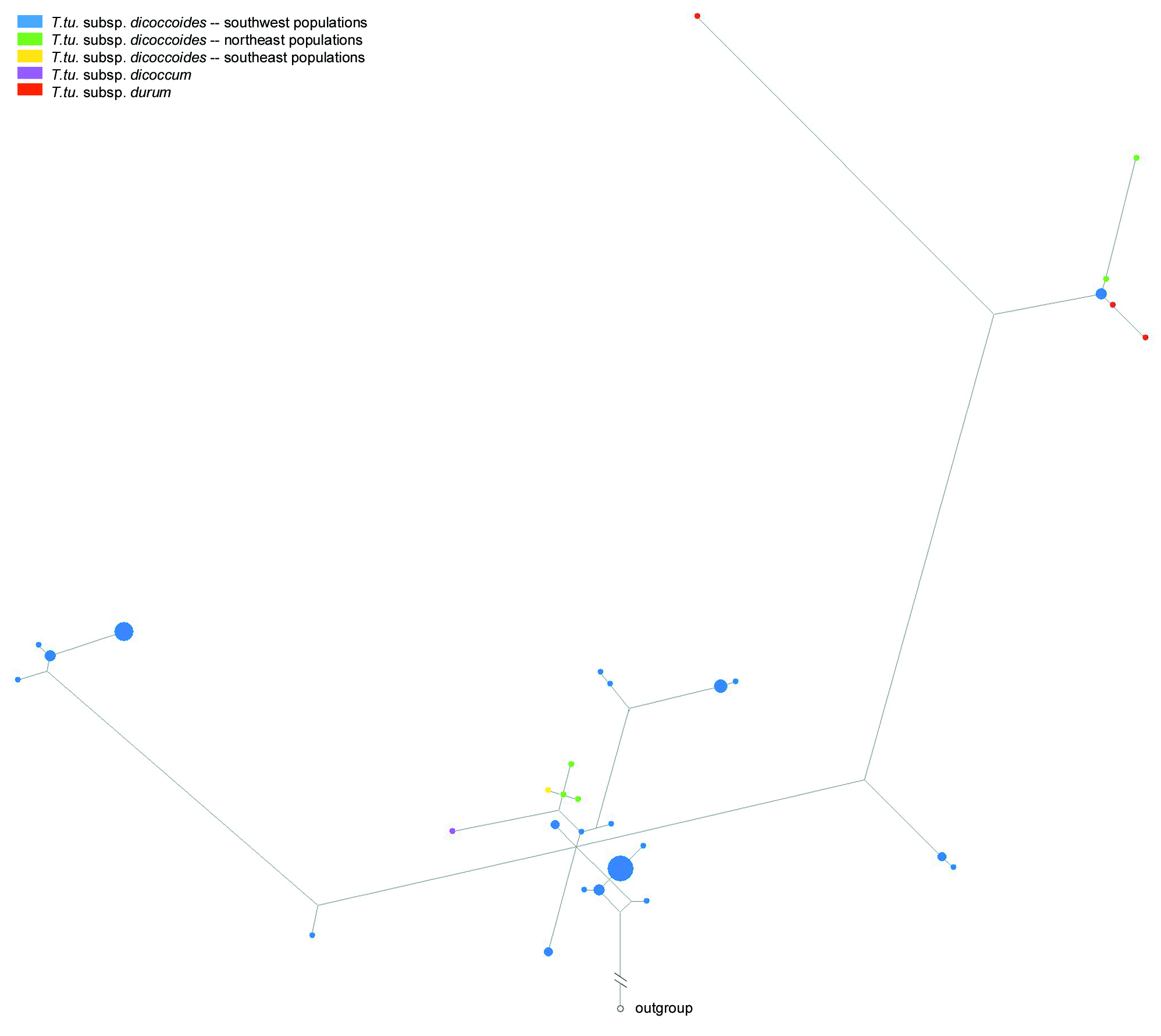


**Fig. D.** Model of the pace of genetic identity disruption after intermixing distinct populations in equal proportions (absence of selection assumed). *n*, number of generations since the populations are mixed; *f(h)*, frequency of individuals with at least one outcrossing event in their genealogy since *n = 0*; *2P*, two populations intermixed; *3P*, three populations intermixed; *f(o)*, frequency of cross-pollination. For two populations, the frequency of hybrids one generation after intermixing equals
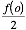
 (half of the cross-pollinating events result in pairing different genotypes). The subsequent rise of *f(h)* is calculated with the formulae
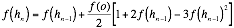
 and
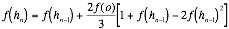
 for *2P* and *3P*, respectively.


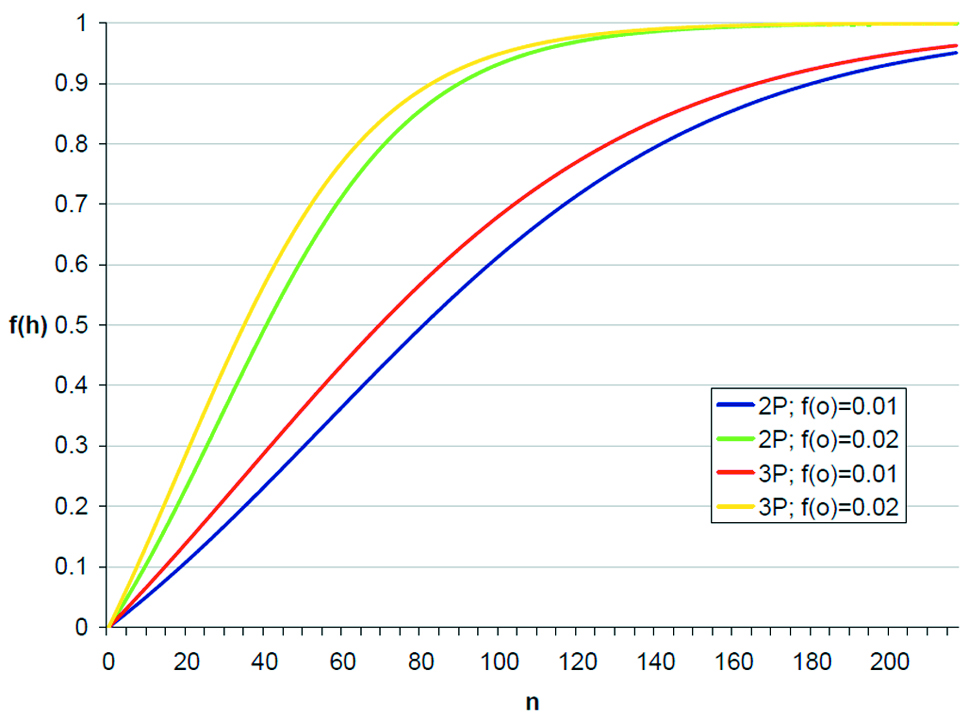


**References**

1. Özkan H, Brandolini A, Schäfer-Pregl R, Salamini F. (2002) AFLP analysis of a collection of tetraploid wheats indicates the origin of emmer and hard wheat domestication in southeast Turkey. Mol Biol Evol 19: 1797–1801.

3. Haudry A, Cenci A, Ravel C, Bataillion T, Brunel D, et al. (2007) Grinding up wheat: a massive loss of nucleotide diversity since domestication. Mol Biol Evol 24: 1506–1517.

4. Feldman M, Kislev ME. (2007) Domestication of emmer wheat and evolution of free-threshing tetraploid wheat. Isr J Plant Sci 55: 207–221.

5. Mori N, Kondo Y, Ishii T, Kawahara T, Valkoun J, Nakamura C. (2009) Genetic diversity and origin of timopheevi wheat inferred by chloroplast DNA fingerprinting. Breed Sci 59: 571–578.

5. Zohary D, Hopf M, Weiss E. (2012) Domestication of plants in the Old World, 4th ed. Oxford: Oxford University Press.

6. Yahiaoui N, Kaur N, Keller B. (2009) Independent evolution of functional *Pm3* resistance genes in wild tetraploid wheat and domesticated bread wheat. Plant J 57: 846–856.
